# Unified Model of Magnetoresistive Angular Position Sensors with Manufacturing Tolerances

**DOI:** 10.3390/s26206435

**Published:** 2026-10-12

**Authors:** Vladimir Čeperković, Vladimir Rajović, Jelena Elez, Milan Prokin, Jelena Popović-Božović

**Affiliations:** 1 Synchrotek d.o.o., Mihajlo Pupin Blvd. 115a, 11070 Belgrade, Serbia; vceperkovic@gmail.com; 2 Department of Electronics, School of Electrical Engineering, University of Belgrade, Bulevar Kralja Aleksandra 73, 11120 Belgrade, Serbia; rajo@etf.bg.ac.rs (V.R.); proka@etf.bg.ac.rs (M.P.); jelena@etf.bg.ac.rs (J.P.-B.)

**Keywords:** physical models, magnetoresistive sensors, anisotropic magnetoresistance, giant magnetoresistance, tunnel magnetoresistance, magnetoresistive measurement bridge, free ferromagnetic layer, angular position sensor, magnetic field direction sensor

## Abstract

This paper presents a unified model of magnetoresistive angular position sensors, applicable to sensors based on the three major sensor technologies: Anisotropic Magnetoresistance, Giant Magnetoresistance, and Tunnel Magnetoresistance magnetoresistive elements. Theoretical models of the three different magnetoresistive elements, including manufacturing tolerances, have been derived first. The models are then employed to derive a realistic model of a magnetoresistive measurement bridge. In order to construct a model of a magnetoresistive magnetic field direction sensor, besides bridge models, a novel model of a free ferromagnetic layer has been developed. Finally, a unified magnetoresistive angular position sensor model has been obtained by combining the magnetic field direction sensor and a simple model of a permanent magnet. The experimental results confirmed the validity of the proposed model.

## 1. Introduction

Magnetoresistive elements represent a broad class of components based on the magnetoresistive effect, which is the measurable change in resistance of certain materials and structures in the presence of a magnetic field. Although the effect was discovered as early as the mid-nineteenth century [1], the first sensor was constructed more than a hundred years later in the form of the Hunt element [2], when their commercial application in magnetic tape heads and for the production of magnetic memories was found [3,4].

High temperature stability and mechanical robustness [5], along with high resolution and accuracy, led to the popularity of magnetoresistive angular position sensors [6], based on the utilization of AMR (Anisotropic Magnetoresistive), GMR (Giant Magnetoresistive), or TMR (Tunnel Magnetoresistive) effects. In such systems, the source of the coupling magnetic field is a permanent magnet mounted on a shaft, whose mechanical position determines the field orientation. The orientation of the magnetic field is then measured by a magnetoresistive field direction sensor. Unfortunately, multiple conversions of the measured quantity, combined with the magnetic field non-homogeneity in the working environment, introduce various sources of measurement errors. The imperfect magnetic field of real magnets, together with mechanical tolerances, results in a periodic measurement error as a function of the mechanical angle [7]. Furthermore, the measurement process is inherently nonlinear due to the non-homogeneity of the magnetic field in ferromagnetic discs [8]. Although these findings were originally reported for Hall sensors with integrated flux concentrators, they are also applicable to magnetoresistive angular position sensors. However, the key aspect of application of magnetoresistive angular position sensors is a precise alignment of the magnet and the sensor, since even subtle deviations lead to unacceptably high measurement error [9].

Existing models of angular position sensor errors typically focus on individual error sources, such as magnetic-field non-uniformity, assembly tolerances, or geometric misalignment, and describe their influence directly in terms of the resulting angular error [7,8,9]. While such models are highly useful for analyzing particular physical mechanisms, they do not generally provide a unified signal-flow representation that separates linear sensor imperfections from the periodic nonlinear angular error. In this work, the complete sensor is represented as a sequence of three functionally distinct segments: a linear segment, a circular modulator, and a nonlinear harmonic segment. This decomposition preserves the relation between physical error sources and the resulting angular error while providing a form suitable for systematic parameter identification, calibration, and compensation.

A magnetoresistive field direction sensor, responsible for converting the angle of the magnetic field induced by the permanent magnet into electrical quantities, consists of two electrically orthogonal magnetoresistive measurement bridges, each composed of multiple magnetoresistive elements. The sensor is connected to an analog conditioning circuit providing either constant voltage or constant current excitation, as well as signal amplification. The intrinsic conversion errors of both the sensor and the conditioning circuit arising from manufacturing tolerances represent a significant contribution to the overall measurement error. However, existing models of magnetoresistive elements are typically derived in idealized form and therefore fail to account for these imperfections. To address this limitation, we propose a parametric formalism that provides a systematic framework for constructing unified models of magnetoresistive sensors with manufacturing tolerances, including a new model of the magnetoresistive element, the magnetoresistive bridge, and the complete field-direction sensor.

The rest of the paper is organized as follows: In Section 2, different magnetoresistivity effects are presented, namely AMR (Section 2.1), GMR (Section 2.2), and TMR (Section 2.3). A novel model of magnetoresistive elements, including manufacturing tolerances are derived in Section 3. Section 4 presents the derivation of a realistic magnetoresistive bridge model, which includes a realistic excitation source (both voltage and current), and a non-ideal instrumentation amplifier. Section 5 deals with modelling of a magnetic field direction sensor, taking into account different operating modes of the free ferromagnetic layer: direct (Section 5.1), linear (Section 5.2), and compensated (Section 5.3). A unified model of a magnetoresistive angular position sensor, utilizing a simple model of a permanent magnet, is developed in Section 6. Section 7 presents experimental verification for AMR sensors (Section 7.1) and simulation-based verification for GMR and TMR sensors (Section 7.2). Concluding remarks are given in Section 8.

## 2. Magnetoresistive Effects

Regardless of the magnetoresistive effect utilized in a particular sensor, its resistance can be described as a function of the magnetization direction in a ferromagnetic layer. In the absence of an external magnetic field, the spontaneous magnetization direction of the free layer coincides with the “easy” axis of the ferromagnetic. According to the coherent rotation model [10], under the influence of an external magnetic field **H**, with component *H_x_* in the direction of the ferromagnetic “easy” axis and the perpendicular component *Hy* in the direction of the ferromagnetic “hard” axis, the magnetization direction of a free layer rotates in the direction of the “hard” axis, so that the angle between the “easy” axis and the magnetization direction, *θ*, satisfies the equilibrium

*H*_0_ ⋅ sin*θ* ⋅ cos*θ* = *H_y_* ⋅ cos*θ* − *H_x_* ⋅ sin*θ*, (1)

where *H*_0_ is a characteristic field of the free ferromagnetic layer. By solving (1), the stable operating region can be determined, and the magnetization angle *θ* can be expressed as

(2) $$\begin{array}{cc} \sin\theta =\frac{H_{y}\cos\theta }{H_{0}\cos\theta +H_{x}}, & \sqrt[{3}]{H_{x}^{2}}+\sqrt[{3}]{H_{y}^{2}}\leq \sqrt[{3}]{H_{o}^{2}}. \end{array}$$

Particularly, when the external magnetic field aligns with the “hard” axis, (2) can be simplified to

(3) $$\begin{array}{cc} \sin\theta =\frac{H_{y}}{H_{o}}, & \left|H_{y}\right|\leq H_{0}. \end{array}$$

Solving any of the above models, i.e., (1), (2), or (3), results in two distinct solutions for the angle *θ*, so the actual solution has to be selected depending on the initial conditions.

### 2.1. Anisotropic Magnetoresistance

The AMR effect occurs in solid conductors composed of 3*d* transition metals (particularly Fe, Co, and Ni) and their alloys (e.g., Ni-Fe permalloy) in the presence of a magnetic field. The relative change in specific resistance *ρ* amounts to 2–4% [11] for commonly used nickel alloys, depending on the purity of the alloy. Phenomenologically, the resistance of a nonlinearized AMR element *R_A_* can be expressed as a function of the angle *γ* between the direction of the element’s magnetization **M** and the electric current **J** through the element (*l*, *w*, and *t* are the length, width and thickness of the element, *R*_0_ is the resistance of the element when *γ* = 90°, and ∆*R_A_* is the resistance change maximum, occurring for *γ* = 0°)

(4) $$\begin{array}{cc} R_{A}\left(\gamma \right) & =\rho \left(\gamma \right)\cdot \frac{l}{w\cdot t}=R_{0}+\Delta R_{A}\cdot \cos^{2}\gamma \\ & =R_{0}+\frac{\Delta R_{A}}{2}+\frac{\Delta R_{A}}{2}\cdot \cos\left(2\gamma \right). \end{array}$$

Of particular importance for the application of the AMR element in measuring the intensity of an external magnetic field is the case when the measured field aligns with the “hard” axis, and the current flow aligns with the “easy” axis. By substituting (3) into (4), a dependence of the resistance of the magnetoresistive element on the intensity of the external magnetic field is obtained

(5) $$\begin{array}{c} R_{A}\left(H\right)=R_{0}+\Delta R_{A}\cdot \left[1-{\left(\frac{H_{y}}{H_{0}}\right)}^{2}\right],\;\left|H_{y}\right|\leq H_{0},\;H_{x}=0. \end{array}$$

Considering that the transfer function (5) exhibits quadratic characteristics, with low sensitivity to weaker magnetic fields and no sensitivity to the direction of the magnetic field, it is beneficial for practical application to adjust the operating point, that is, to appropriately linearize the transfer characteristic [11]. This can be achieved by introducing a bias magnetic field *H_B_* in the direction of the “hard” axis, resulting in a new transfer function

(6) $$\begin{array}{ccc} R_{A}\left(H\right)=\left(R_{0}+\Delta R_{A}\cdot \frac{H_{0}^{2}-H_{B}^{2}}{H_{0}^{2}}\right)-H_{y}\cdot \Delta R_{A}\cdot \frac{2\cdot H_{B}+H_{y}}{H_{0}^{2}}, & \left|H_{y}+H_{B}\right|\leq H_{0}, & H_{x}=0. \end{array}$$

However, magnetic field bias methods are inferior to self-biasing methods, where the element’s shape results in a non-zero angle γ between the electric current and the spontaneous magnetization in the absence of an external magnetic field. In the case of the “herringbone” geometry [12], shape and crystal lattice anisotropy axes are inclined relative to each other so that, in the absence of an external magnetic field, the internal magnetization direction is at an angle *β* relative to the electric current vector through the element. The resistance of such an element can be expressed as

(7) $$\begin{array}{cc} R_{A}\left(\theta \right) & =R_{0}+\Delta R_{A}\cdot \cos^{2}\left(\theta +\beta \right)\\ & =R_{0}+\frac{\Delta R_{A}}{2}+\frac{\Delta R_{A}}{2}\cdot \cos\left(2\cdot \theta +2\cdot \beta \right). \end{array}$$

By choosing appropriate geometric parameters, it is possible to achieve that the angle γ in the absence of an external magnetic field is *β* = ±45°, so that (7) transforms into

(8) $$\begin{array}{cc} R_{A}\left(\theta \right) & =R_{0}+\frac{\Delta R_{A}}{2}\pm \frac{\Delta R_{A}}{2}\cdot \sin\left(2\cdot \theta \right)\\ & =R_{0}+\frac{\Delta R_{A}}{2}\pm \Delta R_{A}\cdot \sin\left(\theta \right)\cdot \cos\left(\theta \right). \end{array}$$

Finally, substituting (3) into (8) yields a linear transfer function in the form

(9) $$\begin{array}{ccc} R_{A}\left(H\right)=R_{0}+\frac{\Delta R_{A}}{2}\pm \Delta R_{A}\cdot \frac{H_{y}}{H_{0}}\cdot \sqrt{1-{\left(\frac{H_{y}}{H_{0}}\right)}^{2}}, & \left|H_{y}\right|\leq H_{0}, & H_{x}=0. \end{array}$$

The sign of the increment is determined by the choice of the geometric configuration. Alternatively, in the “barber pole” self-biasing scheme, the basic element is covered with strips of high-conductivity material placed at an angle of ±45° relative to the “easy” axis of the crystal lattice of the element. The resistance of the conductive strips is significantly lower than the resistance of the ferromagnetic film, which causes the distribution of electric current through the element to be perpendicular to the conductive strips [13]. Therefore, the initial value of the angle γ in the absence of an external magnetic field is ±45°, so the behavior of this element can also be described by (8) and (9).

### 2.2. Giant Magnetoresistance

GMR is a quantum mechanical effect observed in multilayer structures composed of ferromagnetic layers separated by thin conducting nonmagnetic layers. The effect described in [14,15] manifests as a significant change in the resistance of the structure depending on whether the magnetizations in adjacent layers are parallel or antiparallel.

The sandwich structure of a GMR element consists of reference and free thin ferromagnetic film layers, separated by a thin conductive spacer. The magnetization of the reference layer is pinned by a suitable choice of magnetic material or by an additional antiferromagnetic (pinning) layer. Conversely, under the influence of an external magnetic field, the magnetization of the free layer rotates away from the “easy” axis of the layer by an angle *θ*. Considering that the energy density of the interaction between ferromagnetic layers is, in the first approximation, proportional to the scalar product of their magnetizations, it has been shown in [16] that the resistance of the GMR element is given by:

(10) $$R_{G}\left(\theta \right)=\rho \cdot \frac{l}{w\cdot t}\cdot \left(1+\frac{\mathrm{MR}}{2}+\frac{\mathrm{MR}}{2}\cdot \cos\left(\theta -\theta_{p}\right)\right)=R_{0}+\Delta R_{G}\cdot \cos\left(\theta -\theta_{p}\right),$$

where *ρ* represents the effective specific resistance of the element, and the *θ_P_* is the initial angle between “easy” axes of the free and reference layers in the absence of an external magnetic field. The relative change is determined by the magnetoresistive ratio (MR) that, in practical applications, ranges from 4% to 20% for bilayer structures [16], up to 40% for multilayer structures [17].

A linearized GMR element is constructed when the “easy” axes of the free and reference layers are perpendicular to each other. However, in this case up to three independent magnetic fields act along the *y*-axis: external magnetic field *H_y_*, optional polarization magnetic field *H_B_*, and the Néel coupling between the reference and free layers *H_C_* [18]. By taking all those into consideration, and substituting (3) into (10), the dependence of the element’s resistance on the intensity of the external magnetic field is given as:

(11) $$\begin{array}{cc} R_{G}\left(H\right)=R_{0}+\Delta R_{G}\cdot \frac{H_{y}+H_{B}+H_{C}}{H_{0}}, & \left|H_{y}+H_{B}+H_{C}\right|\leq H_{0}. \end{array}$$

The empirical characteristic of the linear GMR element shows good agreement with the theoretical model. Minor deviations occur at the ends of the measurement range due to demagnetization effects in the free layer. In addition, the GMR layered structure exhibits parasitic AMR elements [10] in both ferromagnetic layers, which negatively affects the real transfer characteristic.

### 2.3. TMR Magnetoresistance

TMR effect is a quantum-mechanical phenomenon observed in multilayer structures composed of ferromagnetic layers separated by thin non-conductive non-magnetic layers. The physical basis is the quantum-mechanical tunneling effect of electrons through the potential barrier created by the non-conductive separator [19]. As the electron spin does not change during tunneling, the Mott currents in this case are independent. However, the behavior of the structure can then be modeled using the spin-dependent tunneling method [20]. The tunneling probability for electrons from both electrical currents is higher when the majority spin is identical in both ferromagnetic layers, i.e., when the magnetizations of the layers are parallel.

However, the quantitative difference lies in the achieved magnetoresistive ratios MR, which range from 100% to 200% for practical implementations in the case of the TMR element [21], but exceed 600% in laboratory conditions [22].

## 3. Magnetorezistive Element Model

Considering a multitude of magnetoresistive element realizations, each with its own transfer function, an independent analysis of the behavior of measurement systems based on each of them would be impractical. Instead, the universal characteristic is represented as a function of the free ferromagnetic layer magnetization angle *θ*′, expressed in an electrical coordinate system chosen to eliminate the integer multiplier in the characteristics of AMR elements (4), but without effect on the characteristics of GMR and TMR elements (10), with the initial direction specified by the fabrication parameter *θ_E_*. Further, by noting that the sign of the variable resistance Δ*R_E_* (*θ*′) can be arbitrarily adjusted by a suitable choice of the direction of spontaneous magnetization or geometric parameters of the element, the universal characteristic of the magnetoresistive element *R_E_* (*θ*′) can be expressed in the form:

(12) $$\begin{array}{c} \theta^{\prime }=m_{E}\cdot \theta ,\\ \Delta R_{E}\left(\theta^{\prime }\right)=\Delta R\cdot \cos\left(\theta^{\prime }-\theta_{E}\right),\\ R_{E}\left(\theta^{\prime }\right)=R\pm \Delta R_{E}\left(\theta^{\prime }\right). \end{array}$$

In Table 1, an overview of the parameters *R*, Δ*R*, *θ_E_*, and *m_E_* of the model (12) is provided for common fabrication technologies of integrated magnetoresistive elements.

Next, let ${\hat{R}}_{E}$ (*θ*′) be the resistance of a non-ideal magnetoresistive element as a function of the free ferromagnetic layer magnetization angle *θ’*, the conversion error of the magnetoresistive element *e_E_* (*θ*′), and the idealized model of the magnetoresistive element (12). Then, the model of a real magnetoresistive element can be described as:

(13) $$\begin{array}{cc} {\hat{R}}_{E}\left(\theta^{\prime }\right) & =R_{E}\left(\theta^{\prime }\right)+e_{E}\left(\theta^{\prime }\right)\\ & =R\pm \Delta R\cdot \cos\left(\theta^{\prime }-\theta_{E}\right)+e_{E}\left(\theta^{\prime }\right). \end{array}$$

The conversion errors of integrated magnetoresistive elements in a magnetoresistive sensor are primarily due to technological limitations of the manufacturing process, described in [23,24]. Tolerances during the deposition process affect the thickness and composition of the deposited layer, directly influencing the parameters *R* and Δ*R* of the magnetoresistive element [25]. However, due to the small size of the sensor, each deposited layer can be considered homogeneous, so the parameter model errors are identical for all magnetoresistive elements deposited in the same step. In practice, magnetoresistive elements with the same resistance increment have identical model parameter errors, while for magnetoresistive elements with different resistance increments, this depends on the manufacturing technology. Conversely, tolerances during the photolithography process of the magnetoresistive structure are independent for each magnetoresistive element individually. According to (4) for AMR elements and (10) for GMR and TMR elements, relative tolerances of the magnetoresistive element dimensions equally affect the parameters *R* and Δ*R*. Therefore, it is convenient to model the relative resistance error of the magnetoresistive element arising from geometric thickness tolerances of the deposited magnetoresistive material and tolerances of the dimensions of the magnetoresistive structure formed during the photolithography process with parameter *ε_E_*_,1_, and the relative error of the parameter Δ*R* resulting from tolerances in the composition of the magnetoresistive material with parameter *ε_E_*_,2_.

However, there is no single fabrication step that determines the spontaneous magnetization direction and thus the error of the parameter *θ_E_* of the magnetoresistive element model. Tolerances in the direction of the polarization magnetic field applied during the deposition of the magnetoresistive material cause deviation in the position of the “easy” axis of the crystal lattice. Conversely, tolerances during the photolithography process affect the error in the geometry of the magnetoresistive element, reflecting the deviation of the demagnetizing magnetic field. These deviations affect the direction of the “easy” axis in a complex manner and, consequently, the parameter *θ_E_* of the magnetoresistive element model. Of course, if any of the processes for subsequently establishing the spontaneous magnetization direction are applied during fabrication, their tolerances directly affect the error of this parameter. Therefore, it is convenient to model the combined influence of production tolerances as the absolute error *ε_E_*_,3_ of the parameter *θ_E_* of the magnetoresistive element model.

Finally, when modeling GMR and TMR sensors, an additional angular-dependent resistance contribution associated with the anisotropic magnetoresistance of the ferromagnetic layers may also be present [26]. This parasitic AMR contribution is superimposed on the dominant GMR/TMR response and is represented in the proposed model by an independent parameter. Its magnitude depends on the particular layer structure, materials, and fabrication technology and is therefore treated as an independent model parameter rather than as a fixed universal value. Taking into account that the reference angle of the parasitic AMR element always aligns with the free ferromagnetic layer “easy” axis, and thus does not depend on the characteristics of the reference ferromagnetic layer, the model of the real magnetoresistive element can be described as:

(14) $${\hat{R}}_{E}\left(\theta^{\prime }\right)\approx \left(1+\varepsilon_{E,1}\right)\cdot \left(R\pm \Delta R\cdot \left(1+\varepsilon_{E,2}\right)\cdot \left(\cos\left(\theta^{\prime }-\theta_{E}+\varepsilon_{E,3}\right)+\varepsilon_{E,4}\cdot \cos\left(2\cdot \theta^{\prime }\right)\right)\right).$$

For analytical modeling of the measurement process, the individual manufacturing deviations are grouped into aggregate errors of the parameters of the ideal magnetoresistive element model. The detailed reduction of (14) and the corresponding linearization procedure are provided in Appendix A.1.

Let *r_E_* be the vector of errors of the model parameters of the magnetoresistive element:

(15) $$r_{E}=\left[\begin{array}{c} r_{E,1}\\ r_{E,2}\\ r_{E,3}\\ r_{E,4} \end{array}\right]=\left[\begin{array}{c} \varepsilon_{E,1}\cdot R\\ \left(\varepsilon_{E,1}+\varepsilon_{E,2}+\varepsilon_{E,1}\cdot \varepsilon_{E,2}\right)\cdot \Delta R\\ \varepsilon_{E,3}\\ \left(1+\varepsilon_{E,1}+\varepsilon_{E,2}+\varepsilon_{E,1}\cdot \varepsilon_{E,2}\right)\cdot \varepsilon_{E,4}\cdot \Delta R \end{array}\right].$$

The conversion error of the magnetoresistive element, *e_E_* (*θ*′), in (13) can be approximately determined by applying a first-order linearization around the normal operating point **r***_E_* = 0. Therefore, if *D***_r_** is the operator of the total differential of a function with respect to the elements of the vector **r***_E_*, the conversion error of the magnetoresistive element is approximately given by:

*e_E_*(*θ*′) ≈ *D***_r_***R_E_*(*θ*′) ⋅ **r***_E_*. (16)

Using the differential derived in Appendix A.1 and evaluated at the nominal operating point, the conversion-error model reduces to:

(17) $$\begin{array}{cc} e_{E}\left(\theta^{\prime }\right) & \approx r_{E,1}\\ & +r_{E,2}\cdot \cos\left(\theta^{\prime }-\theta_{E}\right)+r_{E,3}\cdot \Delta R\cdot \sin\left(\theta^{\prime }-\theta_{E}\right)\\ & +r_{E,4}\cdot \cos\left(2\cdot \theta^{\prime }\right). \end{array}$$

Therefore, the conversion error of the free ferromagnetic layer magnetization angle, originating from geometric errors in the photolithographic process, technological errors in the deposition of the magnetoresistive material, and inherent errors of the technology, takes the form of a second-order harmonic function of the free ferromagnetic layer magnetization angle.

## 4. Magnetoresistive Bridge Model

**Figure 1:**
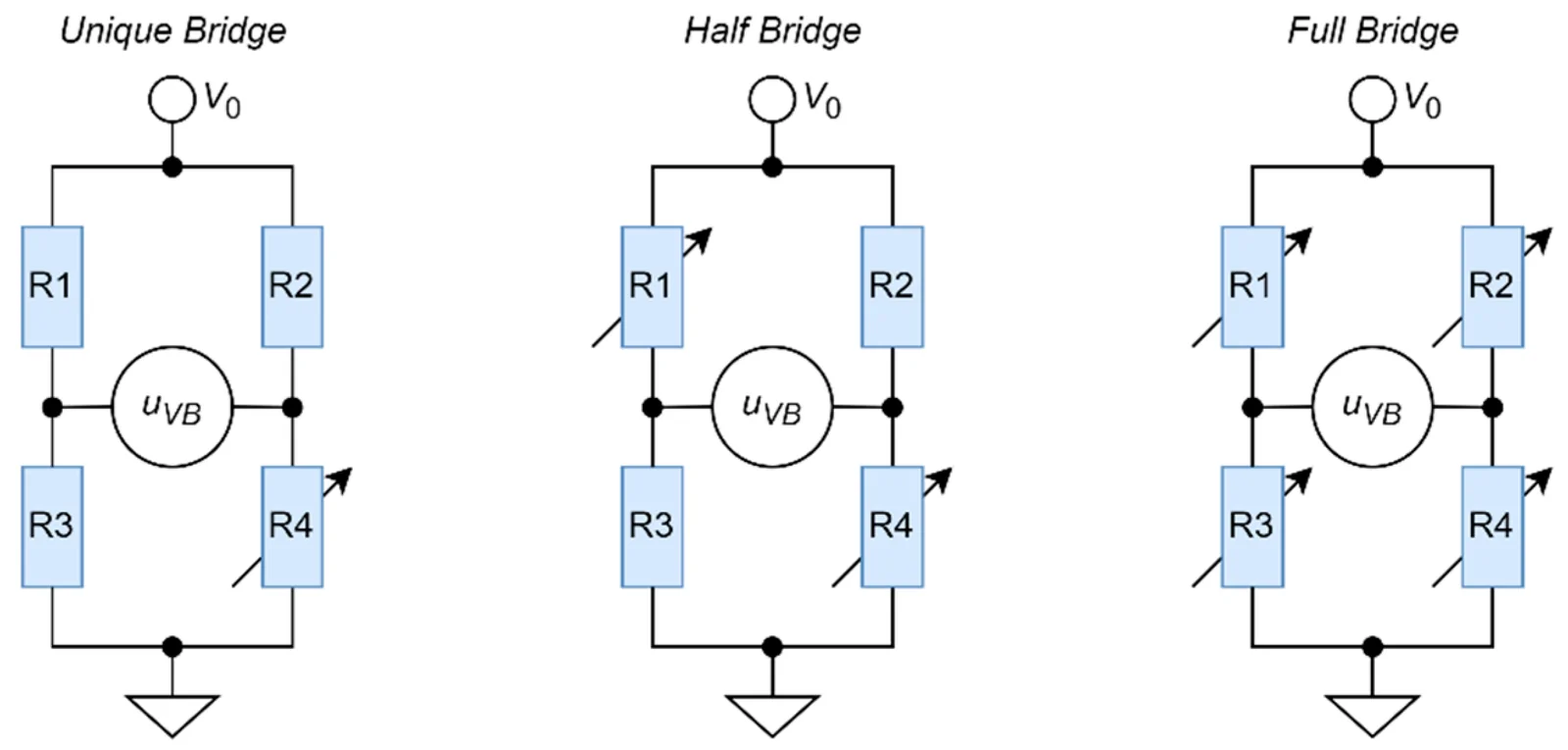
Bridge circuit configurations.

Similarly to most resistive transducers, magnetoresistive elements are also temperature sensitive, as demonstrated in [27] for AMR, [28] for GMR, and [29] for TMR sensors. Specifically, the parameters of the universal characteristic *R* and Δ*R* (12) depend on temperature, with *R* having a positive and Δ*R* a negative temperature coefficient. Therefore, magnetoresistive sensors are almost exclusively fabricated in the form of integrated bridge circuits, which provide inherent temperature compensation. Common configurations of bridge circuits for magnetoresistive sensor fabrication are shown in Figure 1.

Configurations of magnetoresistive measurement bridges with one, two, and four active elements, powered by a constant voltage source (CV) or a constant current source (CC), are well known. Note that inactive resistors in magnetoresistive measurement bridge configurations with one and two active elements are also made as magnetoresistive elements, using the same technique as the active elements. The different behavior is achieved by a protective mask in the form of a flux concentrator made of soft magnetic material [30], which effectively prevents the influence of the external magnetic field. An overview of the nominal resistances of the elements of the magnetoresistive measurement bridge, depending on the angle of spontaneous magnetization and the number of active elements, is given in Table 2.

In the case when the magnetoresistive measurement bridge is powered by a constant voltage source *V*_0_ connected through a linear analog gain interface *A*, the common transfer function of the measurement system is given by:

(18) $$u_{\mathrm{VB}}=A\cdot V_{0}\cdot \left(\frac{R_{4}}{R_{2}+R_{4}}-\frac{R_{3}}{R_{1}+R_{3}}\right).$$

By substituting the values from Table 2 into (18) and analyzing the results, it can be shown that the transfer function of the magnetoresistive measurement bridge powered by a constant voltage source is linear only in the configuration with all four active elements.

On the other hand, when the magnetoresistive measurement bridge is powered by a constant current source *I*_0_ connected through a linear analog gain interface *A*, the transfer function of the measurement system is:

(19) $$u_{\mathrm{CB}}=A\cdot I_{0}\cdot \frac{R_{4}\cdot \left(R_{1}+R_{3}\right)-R_{3}\cdot \left(R_{2}+R_{4}\right)}{R_{1}+R_{2}+R_{3}+R_{4}}.$$

**Figure 2:**
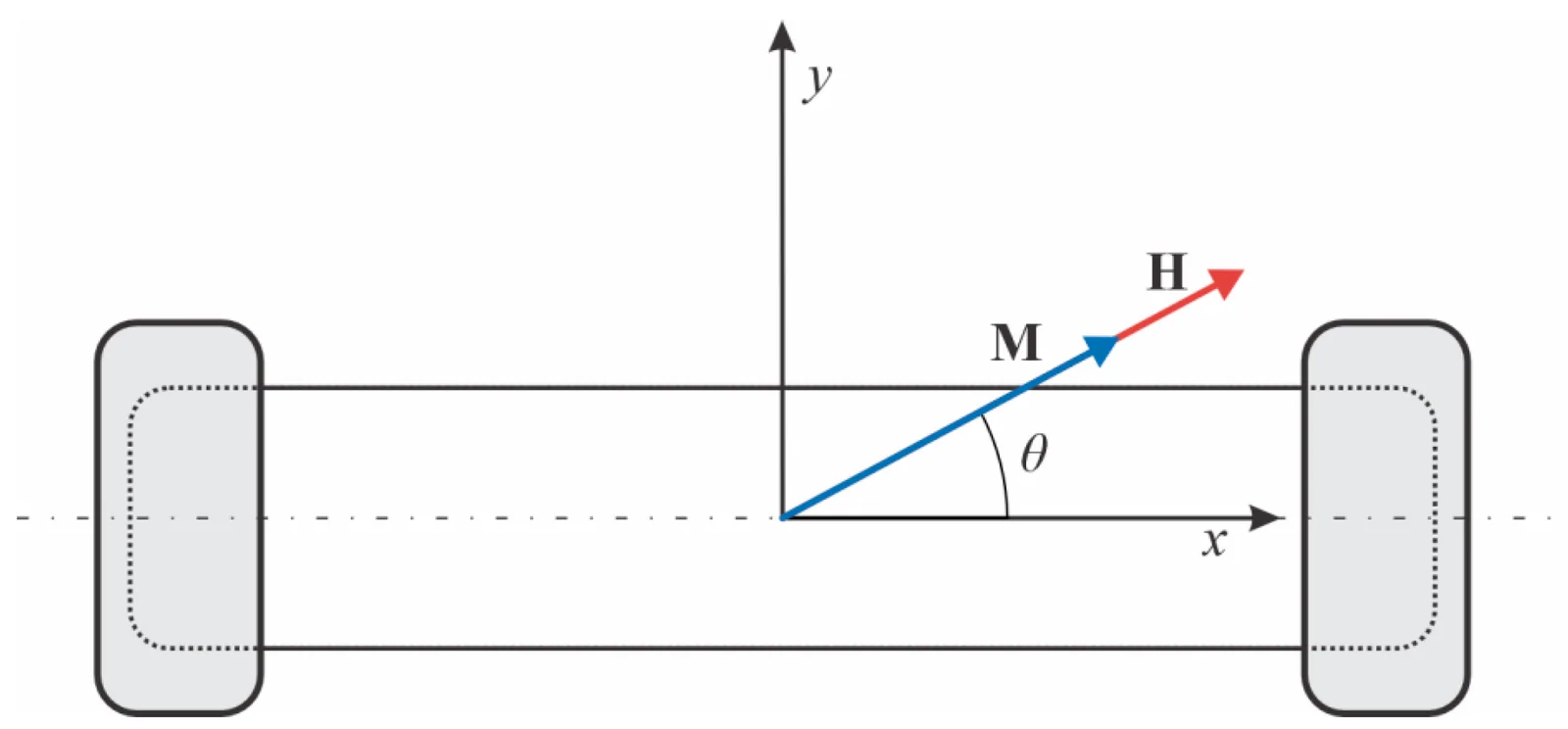
Direct operating modes of the magnetic field direction sensor. The “easy” axis is denoted by dashed line, while the gray boxes represent sensor contacts.

Unlike the magnetoresistive measurement bridge powered by a constant voltage source, by substituting the values from Table 2 into (19), it can be shown that the transfer function of the magnetoresistive measurement bridge powered by a constant current source is linear in configurations with two and four active elements. Since it has been shown that powering with a constant current source reduces the impact of temperature on the bridge parameters, the power supply shown in Figure 2 is now also used for powering magnetoresistive measuring bridges with all four active elements. Regardless of the configuration, all linear magnetoresistive bridges can be described by a linear model with a nominal gain *K_B_*:

*u_B_*(*θ*′) = *K_B_*⋅Δ*R_E_*(*θ*′). (20)

An overview of the parameter *K_B_* values for different configurations of magnetoresistive measuring bridges is provided in Table 3.

Let ${\hat{u}}_{B}$ (*θ*′) be the output of the magnetoresistive measurement bridge model as a function of the free ferromagnetic layer magnetization angle *θ’*, *e_u_* (*θ*′) the measurement error of the measurement bridge, and *u_B_* (*θ*′) the output of the idealized transfer function represented in (20). Then, the model of the real measurement bridge with an analog interface can be described as:

(21) $$\begin{array}{cc} {\hat{u}}_{B}\left(\theta^{\prime }\right) & =u_{B}\left(\theta^{\prime }\right)+e_{u}\left(\theta^{\prime }\right)\\ & =K_{B}\cdot \Delta R_{E}\left(\theta^{\prime }\right)+e_{u}\left(\theta^{\prime }\right). \end{array}$$

The measurement error of the measuring bridge originates from the gain error of the analog interface *g_B_*, the offset error of the analog interface *e*_0_, as well as from the conversion error of the measuring bridge *e_B_* (*θ*′). Therefore, it is:

(22) $$\begin{array}{cc} {\hat{u}}_{B}\left(\theta^{\prime }\right) & =\left(1+g_{B}\right)\cdot \left(K_{B}\cdot \Delta R_{E}\left(\theta^{\prime }\right)+e_{B}\left(\theta^{\prime }\right)+e_{0}\right)\\ & =\left(1+g_{B}\right)\cdot \left(u_{B}\left(\theta^{\prime }\right)+e_{B}\left(\theta^{\prime }\right)+e_{0}\right). \end{array}$$

By comparing (21) with (22), we arrive at the expression for the measurement error of the measuring bridge as a function of the identified error sources:

*e_u_*(*θ*′) = *g_B_* ⋅ *K_B_*⋅Δ*R_E_*(*θ*′) + (1 + *g_B_*) ⋅ (*e_B_*(*θ*′) + *e*_0_). (23)

The conversion error of the measuring bridge *e_B_* (*θ*′) can be approximately determined as a function of the conversion errors of the measuring bridge elements by applying linear approximation to the idealized model (20) around the nominal operating point. Therefore, if **e***_R_* (*θ*′) is the vector of conversion errors of the measuring bridge elements, and *D***_R_** is the operator of the total differential of the function with respect to the resistances of the measuring bridge elements, the conversion error of the measuring bridge is approximately given by:

*e_B_*(*θ*′) ≈ *D***_R_***u_B_*(*θ*′) ⋅ **e***_R_*(*θ*′). (24)

The evaluation of the total differential in (24) depends on the bridge configuration and excitation source. The corresponding derivations for constant-voltage and constant-current excitation are provided in Appendix A.2. Despite the differences between the individual bridge configurations, the resulting total differential at the nominal operating point can be expressed in the common form:

(25) $$\begin{array}{c} D_{R}u_{B}\left(\theta^{\prime }\right)=k_{1}+k_{2}\cdot \frac{\Delta R_{E}\left(\theta^{\prime }\right)}{R},\\ k_{1}=k_{1}\cdot K_{B}\cdot \left[\begin{array}{cccc} 1 & -1 & -1 & 1 \end{array}\right],\\ k_{2}=-k_{2}\cdot K_{B}\cdot \left[\begin{array}{cccc} 1 & 1 & 1 & 1 \end{array}\right]. \end{array}$$

The configuration-dependent parameters **k**_1_ and **k**_2_ of the universal model are summarized in Table 4.

Substituting the common differential form into the linearized model in (24) yields the conversion-error model of the magnetoresistive measurement bridge:

(26) $$e_{B}\left(\theta^{\prime }\right)\approx k_{2}\cdot e_{R}\left(\theta^{\prime }\right)\cdot \frac{\Delta R_{E}\left(\theta^{\prime }\right)}{R}+k_{1}\cdot e_{R}\left(\theta^{\prime }\right).$$

The model also highlights the advantage of constant-current excitation for bridges with four active elements. For identical bridge gains *K_B_*, the constant-current configuration eliminates the contribution associated with **k**_2_, whereas under constant-voltage excitation the relative contribution of the **k**_2_- and **k**_1_-dependent terms is proportional to the magnetoresistive ratio *MR*. Consequently, constant-current excitation is particularly advantageous for AMR and GMR sensors, while TMR sensors require a more detailed analysis.

To express the bridge conversion error in (26) explicitly as a function of the free ferromagnetic layer magnetization angle, the individual magnetoresistive-element error model is substituted into the bridge error formulation. The resulting terms can be rearranged into a finite set of harmonic functions of the magnetization angle. The detailed matrix formulation, trigonometric transformations, and the corresponding mapping between the model coefficients and harmonic functions are provided in Appendix A.2. Consequently, the conversion error of the magnetoresistive measurement bridge can be expressed as a harmonic series of up to the third order:

(27) $$\begin{array}{cc} e_{B}\left(\theta^{\prime }\right) & \approx \sum_{i=1}^{8}e_{B,i}\cdot f_{i}\left(\theta^{\prime }\right)\\ & =\underset{\mathrm{order}=0}{\underset{︸}{e_{B,1}}}+\underset{\mathrm{order}=1}{\underset{︸}{\sum_{i=2}^{4}e_{B,i}\cdot f_{i}\left(\theta^{\prime }\right)}}+\underset{\mathrm{oreder}=2}{\underset{︸}{\sum_{i=5}^{7}e_{B,i}\cdot f_{i}\left(\theta^{\prime }\right)}}+\underset{\mathrm{oreder}=3}{\underset{︸}{e_{B,8}\cdot f_{8}\left(\theta^{\prime }\right)}}. \end{array}$$

The coefficients *e_B,i_* and the corresponding harmonic functions *f_i_* are provided in Table A1 of Appendix A.2.

According to (27), the conversion error of the magnetoresistive measurement bridge is described by a harmonic function of the free ferromagnetic layer magnetization angle, up to the third order at most. However, according to Table 4, the parameter *k*_2_ is always equal to zero in the case of a measurement bridge powered by a constant current source, so based on the expression from Table A1 of Appendix A.2, in this case the coefficients *e_B_*_,6_, *e_B_*_,7_, and *e_B_*_,8_ are all zero. Therefore, when the measurement bridge is powered by a constant current source, the conversion error is described by a harmonic function up to the second order at most.

Regardless of the power source, the order of the harmonic function (27) is always higher for measurement bridges implemented using GMR or TMR elements than for measurement bridges implemented using AMR elements. Specifically, parameter *r_S_*_,4_ models the parasitic AMR effect in GMR and TMR elements, while its value is zero in AMR element models. Therefore, in the model of magnetoresistive measurement bridges based on AMR elements, there are no harmonic terms with coefficients *e_B_*_,5_ and *e_B_*_,8_, which makes the harmonic function (27) second order in the case of a measurement bridge powered by a constant voltage source, or first order in the case of a measurement bridge powered by a constant current source.

Finally, combining the harmonic representation in (27) with the universal magnetoresistive-element characteristic yields the following model of the magnetoresistive measurement bridge:

(28) $$\begin{array}{cc} {\hat{u}}_{B}\left(\theta^{\prime }\right) & =\left(1+g_{B}\right)\cdot K_{B}\cdot \Delta R\cdot f_{2}\left(\theta^{\prime }\right)+\left(1+g_{B}\right)\cdot \left(\sum_{i=1}^{8}e_{B,i}\cdot f_{i}\left(\theta^{\prime }\right)+e_{0}\right)\\ & =\underset{\mathrm{order}=0}{\underset{︸}{\left(1+g_{B}\right)\cdot \left(e_{B,1}+e_{0}\right)}}+\underset{\mathrm{order}=1}{\underset{︸}{\left(1+g_{B}\right)\cdot \left(K_{B}\cdot \Delta R\cdot f_{2}\left(\theta^{\prime }\right)+\sum_{i=2}^{4}e_{B,i}\cdot f_{i}\left(\theta^{\prime }\right)\right)}}\\ & +\underset{1<\mathrm{order}\leq 3}{\underset{︸}{\left(1+g_{B}\right)\cdot \sum_{i=5}^{8}e_{B,i}\cdot f_{i}\left(\theta^{\prime }\right)}}. \end{array}$$

The model of the magnetoresistive measurement bridge (28) is a harmonic function of the free ferromagnetic layer magnetization angle, up to the third order at most. The actual order of the harmonic function in the model depends on the choice of the measurement bridge power supply, as well as the choice of magnetoresistive element technology, in the same way as the measurement bridge error model depends on the angle of spontaneous magnetization of the free ferromagnetic layer (27).

## 5. Magnetic Field Direction Sensor Model

A magnetoresistive sensor for measuring the direction of a magnetic field consists of two integrated magnetoresistive bridge circuits oriented so that their transfer functions are phase-shifted by 90°. Therefore, the output *u_x_* (*ϕ*) of the first bridge circuit is proportional to the cosine of the angle of the external magnetic field *ϕ*, while the output *u_y_* (*ϕ*) of the second bridge circuit is proportional to the sine of the angle of the external magnetic field *ϕ*. Together, these two signals form the measurement vector of the magnetic field direction **u** (*ϕ*) in the sensor’s electrical coordinate system.

The magnetic field direction sensor can be designed to operate in three basic operational modes: direct, linear, or compensated. The specific operational mode is determined by the intensity of the external magnetic field *H* relative to the sensor’s characteristic magnetic field intensity *H*_0_, according to the following criteria [31]:

- *H* ≫ *H*_0_, Direct Operating Mode
- *H* ≪ *H*_0_, Linear Operating Mode
- *H* ≈ 0, Compensated Operating Mode

Therefore, the relative intensity of the external magnetic field *H* compared to the characteristic magnetic field intensity of the sensor *H*_0_ qualitatively changes the sensor behavior.

### 5.1. Direct Operating Mode

When the external magnetic field intensity is significantly higher than the characteristic magnetic field of the sensor, the magnetic field direction sensor operates in the direct mode, depicted in Figure 2 in the electrical coordinate system of the sensor.

In this case, the spontaneous magnetization of the free ferromagnetic layer **M** saturates, so the intensity of the external magnetic field *H* does not affect the sensor’s state. On the other hand, the direction of the spontaneous magnetization *θ* of the free ferromagnetic layer aligns with the direction of the external magnetic field *ϕ*. By substituting (12) into (A1) independently for the *x* and *y* branches of the bridge, and considering the described geometric relationships, we arrive at the transfer function of the magnetic field direction sensor in the direct operating mode:

(29) $$\begin{array}{c} u_{x}\left(\phi \right)=K_{B}\cdot \Delta R\cdot \cos\left(m_{E}\cdot \phi \right),\\ u_{y}\left(\phi \right)=K_{B}\cdot \Delta R\cdot \sin\left(m_{E}\cdot \phi \right). \end{array}$$

Let us adopt *θ*_0_ as the reference angle of the magnetic field direction sensor in the electrical coordinate system of the sensor. Furthermore, let ϕ′ be the direction of the external magnetic field in the electrical coordinate system, such that:

*ϕ*′ = *m_E_* ⋅ *ϕ* = *θ*′ + *θ*_0_. (30)

By replacing (30) into the measurement bridge model (28), we arrive at the model of the measuring bridge as a function of the angle of the external magnetic field, as follows:

(31) $$\begin{array}{cc} {\hat{u}}_{B}\left(\phi^{\prime }\right) & =\underset{\mathrm{order}=0}{\underset{︸}{\left(1+g_{B}\right)\cdot \left(e_{B,1}+e_{0}\right)}}+\underset{\mathrm{order}=1}{\underset{︸}{\left(1+g_{B}\right)\cdot \left(K_{B}\cdot \Delta R\cdot f_{2}\left(\phi^{\prime }-\theta_{0}\right)+\sum_{i=2}^{4}e_{B,i}\cdot f_{i}\left(\phi^{\prime }-\theta_{0}\right)\right)}}\\ & +\underset{\mathrm{order}>1}{\underset{︸}{\left(1+g_{B}\right)\cdot \sum_{i=5}^{7}e_{B,i}\cdot f_{i}\left(\phi^{\prime }-\theta_{0}\right)}}. \end{array}$$

Let us consider the first-order harmonic term in (31). Notice that it contains the entire transfer function of the ideal measuring bridge (20), as well as the influence of the measuring bridge conversion error and the error of the analog interface. Therefore, it is convenient to represent this term in an alternative form by introducing the equivalent amplification of the measuring bridge *A_B_* and the equivalent phase shift in the measuring bridge *θ_B_* such that:

(32) $$A_{B}\cdot \cos\left(\phi^{\prime }-\theta_{0}+\theta_{B}\right)=\left(1+g_{B}\right)\cdot \left(K_{B}\cdot \Delta R\cdot f_{2}\left(\phi^{\prime }-\theta_{0}\right)+\sum_{i=2}^{4}e_{B,i}\cdot f_{i}\left(\phi^{\prime }-\theta_{0}\right)\right).$$

Let us introduce the equivalent offset *o_B_* of the measuring bridge model as a derived parameter according to:

*o_B_* = (1 + *g_B_*) ⋅ (*e_B_*_,1_ + *e*_0_). (33)

Finally, by substituting (32) and (33) into (22), we arrive at the model of the measuring bridge in the direct operating mode:

(34) $${\hat{u}}_{B}\left(\theta^{\prime }\right)=A_{B}\cdot \cos\left(\phi^{\prime }-\theta_{0}+\theta_{B}\right)+o_{B}+\left(1+g_{B}\right)\cdot \sum_{i=5}^{8}e_{B,i}\cdot f_{i}\left(\phi^{\prime }-\theta_{0}\right).$$

Without loss of generality, let us adopt the reference direction of the magnetic field sensor such that it aligns with the actual direction of the magnetoresistive measuring bridge located along the y-axis:

*ϕ*′ = *θ*′ + *θ_y_*. (35)

By substituting the real model of the measuring bridge (34) independently for bridges along the *x* and *y* axes, considering the adopted reference direction (35) and the described geometric relationships, we arrive at the combined model of the magnetic field direction sensor bridges in the direct operating mode:

(36) $$\begin{array}{c} {\hat{u}}_{x}\left(\phi^{\prime }\right)=A_{x}\cdot \cos\left(\phi^{\prime }+\theta_{x}-\theta_{y}\right)+o_{x}+\left(1+g_{x}\right)\cdot \sum_{i=5}^{8}e_{x,i}\cdot f_{i}\left(\phi^{\prime }-\theta_{y}\right),\\ {\hat{u}}_{y}\left(\phi^{\prime }\right)=A_{y}\cdot \cos\left(\phi^{\prime }-\frac{\pi }{2}\right)+o_{y}+\left(1+g_{y}\right)\cdot \sum_{i=5}^{8}e_{y,i}\cdot f_{i}\left(\phi^{\prime }-\theta_{y}-\frac{\pi }{2}\right). \end{array}$$

The model (36) can be conveniently represented in matrix notation:

(37) $$\hat{u}\left(\phi^{\prime }\right)=G\cdot \left[\begin{array}{c} \cos\left(\phi^{\prime }\right)\\ \sin\left(\phi^{\prime }\right) \end{array}\right]+o+e\left(\phi^{\prime }\right),$$

where the gain matrix of the model **G**, phase shift ϕ_0_, and offset vector of the model o are given as:

(38) $$\begin{array}{ccc} G=\left[\begin{array}{cc} A_{x}\cdot \cos\phi_{0} & -A_{x}\cdot \sin\phi_{0}\\ 0 & A_{y} \end{array}\right], & \phi_{0}=\theta_{x}-\theta_{y}, & o=\left[\begin{array}{c} o_{x}\\ o_{y} \end{array}\right], \end{array}$$

while the residual error **e** (ϕ’) is given by:

(39) $$e\left(\phi^{\prime }\right)=\left[\begin{array}{c} e_{x}\left(\phi^{\prime }\right)\\ e_{y}\left(\phi^{\prime }\right) \end{array}\right]=\left[\begin{array}{c} \left(1+g_{x}\right)\cdot \sum_{i=5}^{8}e_{x,i}\cdot f_{i}\left(\phi^{\prime }-\theta_{y}\right)\\ \left(1+g_{y}\right)\cdot \sum_{i=5}^{8}e_{y,i}\cdot f_{i}\left(\phi^{\prime }-\theta_{y}-\frac{\pi }{2}\right) \end{array}\right].$$

The magnetoresistive magnetic field direction sensor is described using the gain matrix model **G**, offset vector model **o**, and harmonic residual error model **e** (*ϕ*′) in terms of the direction of the external magnetic field in the electrical coordinate system.

The residual error model **e** (*ϕ*′) contains only second- and third-order terms, depending on the choice of the measurement bridge power supply, as well as the choice of magnetoresistive element technology. Thus, the residual error model of the magnetoresistive measurement bridge with AMR elements powered by a constant current source contains no terms. Conversely, the residual error model contains a third-order term only in the case of a magnetoresistive measurement bridge with GMR or TMR elements powered by a constant voltage source, which is due to the coefficients *e_x_*_,8_ and *e_y_*_,8_ alongside the third-order harmonic function *f*_8_, which models the parasitic AMR sensor.

According to (32), the derived parameters (38) depend on physical quantities with significant temperature sensitivity, primarily the offset and gain of the analog interface, as well as the offset and sensitivity of the magnetoresistive measurement bridge. Conversely, according to Table 5, the temperature sensitivity of the residual error model (39) may also depend on the temperature dependence of the parasitic AMR contribution in GMR/TMR implementations.

### 5.2. Linear Operating Mode

**Figure 3:**
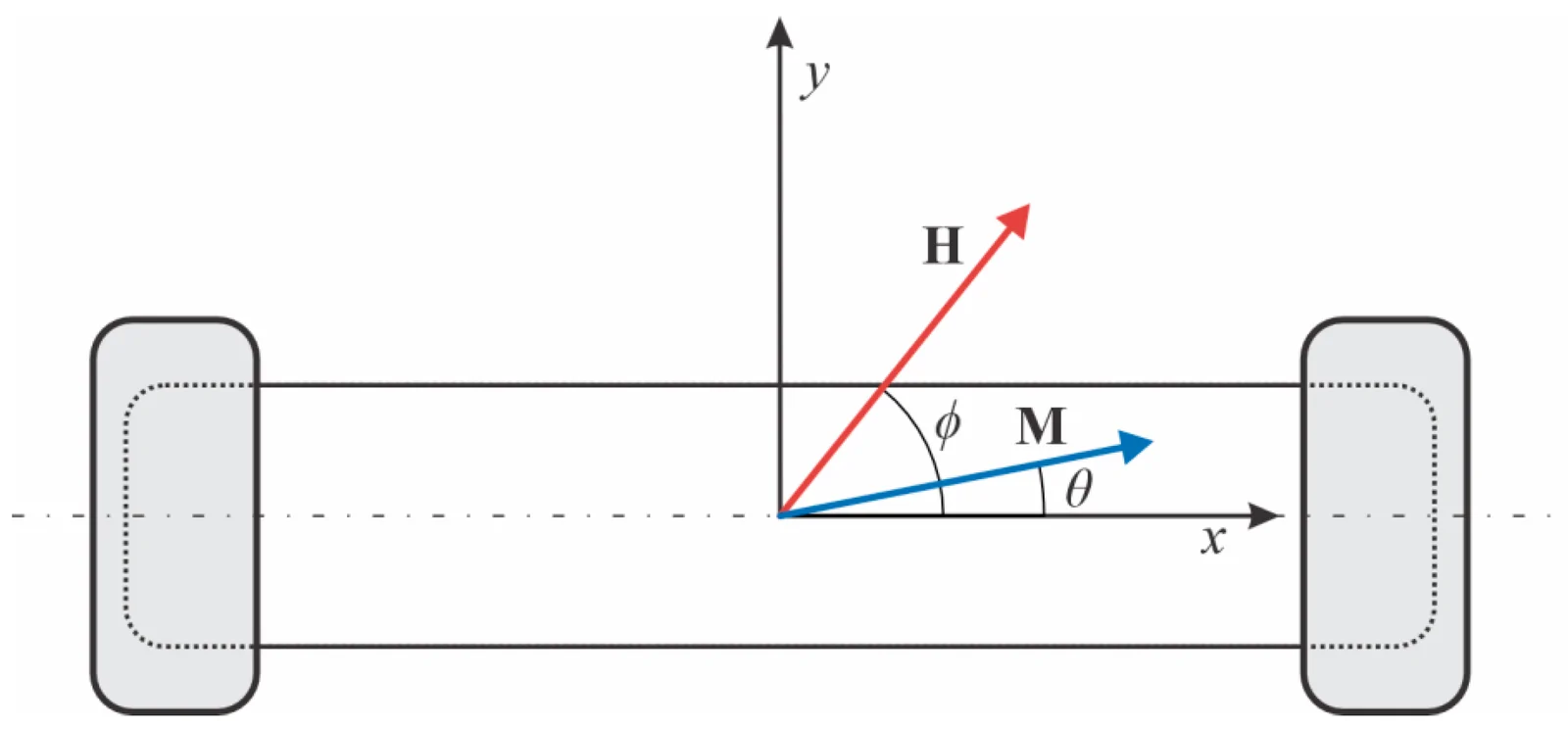
Linear operating modes of the magnetic field direction sensor. The “easy” axis is denoted by dashed line, while the gray boxes represent sensor contacts.

When the external magnetic field intensity is significantly lower than the characteristic magnetic field of the sensor, the magnetic field direction sensor operates in the linear mode, depicted in Figure 3 in the electrical coordinate system of the sensor.

In the linear operating mode, the free ferromagnetic layer magnetization is a function of both the intensity and direction of the external magnetic field as described (1), which in a slightly modified form reads:

(40) $$\sin\theta =\frac{H_{y}}{H_{0}}\cdot \frac{1}{1+\frac{H_{x}}{H_{0}\cdot \cos\theta }}.$$

However, since the intensity of the external magnetic field is significantly smaller than the characteristic magnetic field, the angle of spontaneous magnetization of the free ferromagnetic layer is relatively small, with maximum amplitudes ranging from 2° to 5°. Therefore, it can be assumed that *cos θ* ≈ 1 and, after some rearranging, that:

(41) $$\sin\left(m_{E}\cdot \theta \right)\approx m_{E}\cdot \frac{H}{H_{0}}\cdot \sin\phi .$$

Furthermore, by substituting (41) and (12) into (20) independently for the *x* and *y* branches of the bridge, and considering the described geometric relations, we obtain the transfer function of the magnetic field direction sensor in the linear operating mode:

(42) $$\begin{array}{l} u_{x}\left(\phi \right)=\frac{K_{B}\cdot \Delta R\cdot m_{E}}{H_{0}}\cdot H\cdot \cos\phi ,\\ u_{y}\left(\phi \right)=\frac{K_{B}\cdot \Delta R\cdot m_{E}}{H_{0}}\cdot H\cdot \sin\phi . \end{array}$$

For the application of the linear operating regime of the magnetic field direction sensor, it is necessary for all magnetoresistive elements to be linear. Therefore, based on Table 1, the parameter *θ_E_* is always 90° regardless of the magnetoresistive element technology used. To determine the model, it is necessary to further determine the harmonic function *f*_7_ depending on the angle of the magnetic field, which can be achieved using the approximation (41):

(43) $$\begin{array}{cc} f_{7}\left(m_{E}\cdot \theta \right) & =\sin\left(2\cdot m_{E}\cdot \theta -2\cdot \theta_{E}\right)=-\sin\left(2\cdot m_{E}\cdot \theta \right)\\ & =-2\cdot \sin\left(m_{E}\cdot \theta \right)\cdot \cos\left(m_{E}\cdot \theta \right)\\ & \approx -2\cdot \frac{m_{E}\cdot H}{H_{0}}\cdot \sin\phi . \end{array}$$

Similarly, the harmonic function *f*_8_ depending on the angle of the magnetic field can be determined as follows:

(44) $$\begin{array}{cc} f_{8}\left(m_{E}\cdot \theta \right) & =\cos\left(3\cdot m_{E}\cdot \theta -\theta_{E}\right)=\sin\left(3\cdot m_{E}\cdot \theta \right)\\ & =3\cdot \sin\left(m_{E}\cdot \theta \right)-4\cdot \sin^{3}\left(m_{E}\cdot \theta \right)\\ & \approx 3\cdot \frac{m_{E}\cdot H}{H_{0}}\cdot \sin\phi -4\cdot {\left(\frac{m_{E}\cdot H}{H_{0}}\right)}^{3}\cdot \sin^{3}\phi \\ & =3\cdot \frac{m_{E}\cdot H}{H_{0}}\cdot \sin\phi -4\cdot {\left(\frac{m_{E}\cdot H}{H_{0}}\right)}^{3}\cdot \frac{3\cdot \sin\phi -\sin\left(3\cdot \phi \right)}{4}\\ & =3\cdot \frac{m_{E}\cdot H}{H_{0}}\cdot \left(1-{\left(\frac{m_{E}\cdot H}{H_{0}}\right)}^{2}\right)\cdot \sin\phi +{\left(\frac{m_{E}\cdot H}{H_{0}}\right)}^{3}\cdot \sin\left(3\cdot \phi \right). \end{array}$$

Based on the obtained results, it is now possible to present all harmonic functions *f_i_* for the application of linear magnetoresistive elements, which are summarized in Table 5.

By substituting the expressions from Table 5 into the measuring bridge model (28), we arrive at the model of the measuring bridge as a function of the angle of the external magnetic field in the linear regime, as follows:

(45) $$\begin{array}{cc} {\hat{u}}_{B}\left(\phi \right) & =\underset{\mathrm{order}=0}{\underset{︸}{\left(1+g_{B}\right)\cdot \left(e_{B,1}-e_{B,3}+e_{B,5}-e_{B,6}+e_{0}\right)}}\\ & +\underset{\mathrm{order}=1}{\underset{︸}{\left(1+g_{B}\right)\cdot \frac{m_{E}\cdot H}{H_{0}}\cdot \left(K_{B}\cdot \Delta R+e_{B,2}-e_{B,4}-e_{B,7}+3\cdot e_{B,8}\cdot \left(1-{\left(\frac{m_{E}\cdot H}{H_{0}}\right)}^{2}\right)\right)\cdot \sin\phi }}\\ & +\underset{\mathrm{order}=3}{\underset{︸}{\left(1+g_{B}\right)\cdot e_{B,8}\cdot {\left(\frac{m_{E}\cdot H}{H_{0}}\right)}^{3}\cdot \sin\left(3\cdot \phi \right)}}. \end{array}$$

By repeating the same method as employed in the direct regime, let us determine the equivalent gain of the measuring bridge model *A_B_* and the equivalent offset *o_B_* of the measuring bridge model, according to:

(46) $$\begin{array}{c} A_{B}=\left(1+g_{B}\right)\cdot \frac{m_{E}\cdot H}{H_{0}}\cdot \left(K_{B}\cdot \Delta R+e_{B,2}-e_{B,4}-e_{B,7}+3\cdot e_{B,8}\cdot \left(1-{\left(\frac{m_{E}\cdot H}{H_{0}}\right)}^{2}\right)\right),\\ o_{B}=\left(1+g_{B}\right)\cdot \left(e_{B,1}-e_{B,3}+e_{B,5}-e_{B,6}+e_{0}\right). \end{array}$$

Finally, by substituting (46) into (45), we arrive at the model of the measuring bridge in the linear operating regime:

(47) $${\hat{u}}_{B}\left(\theta \right)=A_{B}\cdot \sin\phi +o_{B}+\left(1+g_{B}\right)\cdot e_{B,8}\cdot {\left(\frac{m_{E}\cdot H}{H_{0}}\right)}^{3}\cdot \sin\left(3\cdot \phi \right).$$

Again, let us adopt the actual direction of the magnetoresistive measuring bridge located along the *y*-axis as the reference direction for the magnetic field direction sensor. By substituting the measuring bridge model in the linear operating regime (47) independently for the measuring bridges located along the *x* and *y* axes, considering the adopted reference direction and the described geometric relationships, we arrive at the combined model of the magnetic field direction sensor bridges in the linear operating regime:

(48) $$\begin{array}{l} {\hat{u}}_{x}\left(\phi \right)=A_{x}\cdot \cos\left(\phi +\phi_{0}\right)+o_{x}+\left(1+g_{x}\right)\cdot e_{x,8}\cdot {\left(\frac{m_{E}\cdot H}{H_{0}}\right)}^{3}\cdot \cos\left(3\cdot \left(\phi +\phi_{0}\right)\right),\\ {\hat{u}}_{y}\left(\phi \right)=A_{y}\cdot \sin\phi +o_{y}+\left(1+g_{y}\right)\cdot e_{y,8}\cdot {\left(\frac{m_{E}\cdot H}{H_{0}}\right)}^{3}\cdot \sin\left(3\cdot \phi \right). \end{array}$$

The model (48) can be conveniently represented in matrix notation:

(49) $$\hat{u}\left(\phi \right)=G\cdot \left[\begin{array}{c} \cos\phi \\ \sin\phi \end{array}\right]+o+e\left(\phi \right),$$

where the gain matrix of the model **G** and the offset vector of the model **o** are given by:

(50) $$\begin{array}{cc} G=\left[\begin{array}{cc} A_{x}\cdot \cos\phi_{0} & -A_{x}\cdot \sin\phi_{0}\\ 0 & A_{y} \end{array}\right], & o=\left[\begin{array}{c} o_{x}\\ o_{y} \end{array}\right], \end{array}$$

while the residual error **e** (*θ*) is given by:

(51) $$e\left(\phi \right)=\left[\begin{array}{c} e_{x}\left(\phi \right)\\ e_{y}\left(\phi \right) \end{array}\right]=\left[\begin{array}{c} \left(1+g_{x}\right)\cdot e_{x,8}\cdot {\left(\frac{m_{E}\cdot H}{H_{0}}\right)}^{3}\cdot \cos\left(3\cdot \left(\phi +\phi_{0}\right)\right)\\ \left(1+g_{y}\right)\cdot e_{y,8}\cdot {\left(\frac{m_{E}\cdot H}{H_{0}}\right)}^{3}\cdot \sin\left(3\cdot \phi \right) \end{array}\right].$$

It is interesting to note that the gain matrix of the model **G** in the case of the linear operating regime is structurally identical to the gain matrix of the model **G** in the direct operating regime. Similarly, the phase shift *ϕ*_0_ is identical, which is an expected result considering that it is predominantly determined by the sensor’s geometry. Conversely, the residual error **e** (*ϕ*) in the linear operating regime exists only in the case of a magnetoresistive measurement bridge with GMR or TMR elements powered by a constant voltage source, where it reduces to a third-order term due to parasitic AMR elements.

### 5.3. Compensated Operating Mode

**Figure 4:**
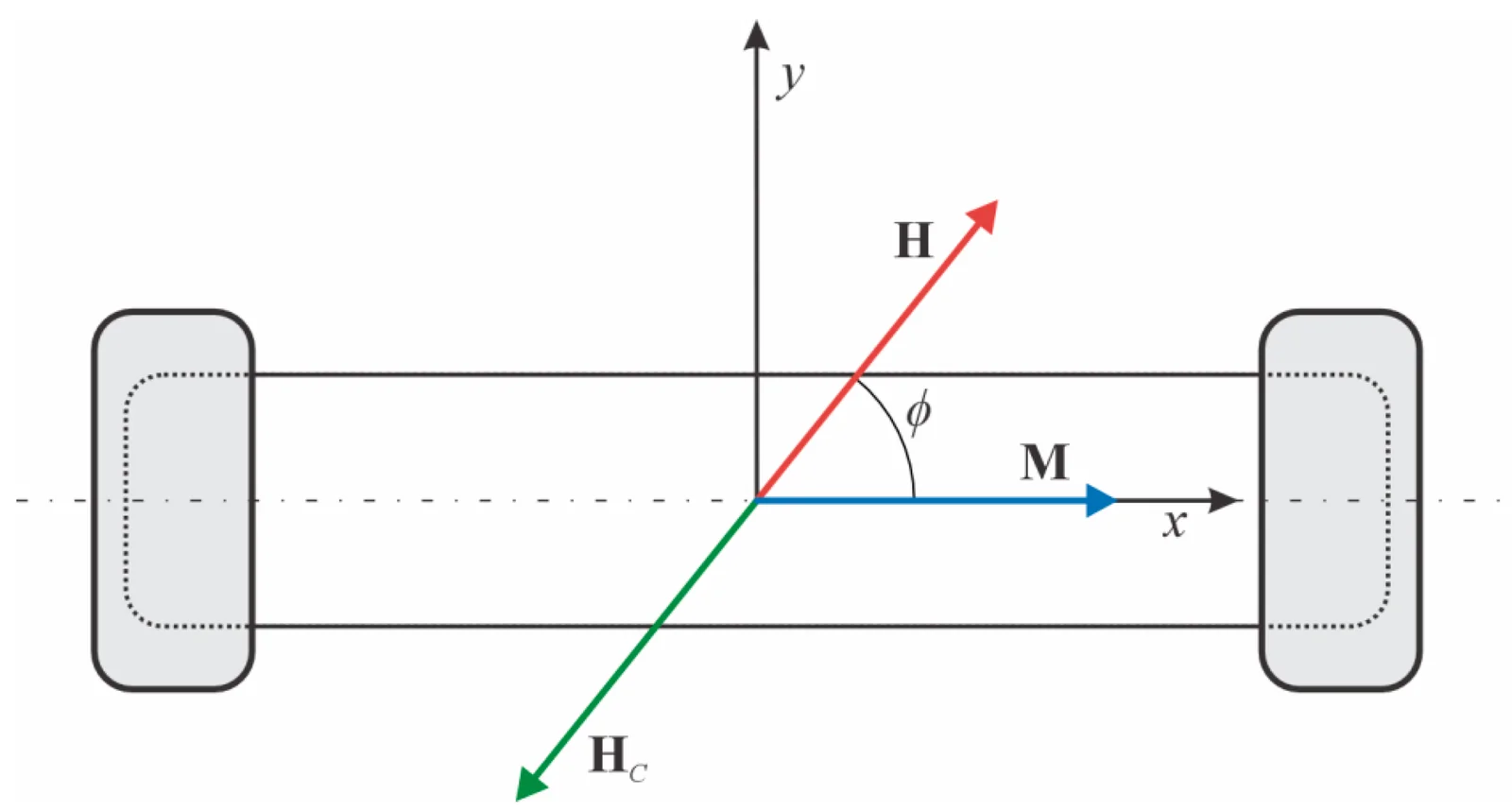
Compensated operating modes of the magnetic field direction sensor. The “easy” axis is denoted by dashed line, while the gray boxes represent sensor contacts.

Finally, Figure 4 illustrates the compensated operating mode of the magnetic field direction sensor.

Unlike the previously mentioned operating modes, in this case, the coordinate system is determined by the compensation windings, and thus it usually coincides with the mechanical one. Therefore, when the magnetic field direction sensor is in the compensated operating mode, the external magnetic field is compensated by the application of independent electromagnets. The analog interface forms a feedback loop, independently for each axis, ensuring that the bridge circuits are balanced. At the same time, the output of such a sensor is the control signal for the electromagnets, with the amplification provided by the analog interface *A*, as follows:

(52) $$\begin{array}{l} u_{x}\left(\phi \right)=A\cdot H\cdot \cos\phi ,\\ u_{y}\left(\phi \right)=A\cdot H\cdot \sin\phi . \end{array}$$

**Figure 5:**
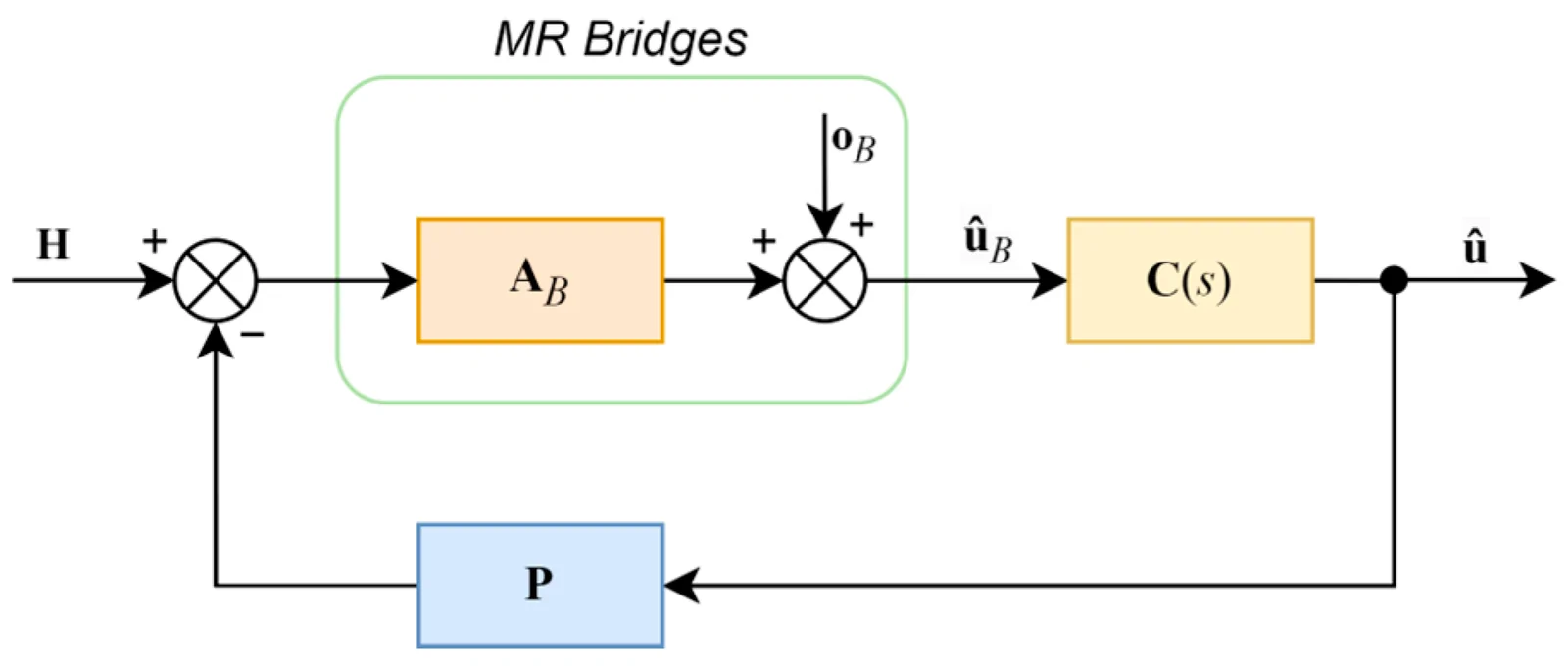
Signal flow diagram of the magnetic field direction sensor in the compensated mode.

The combined model of the magnetic field direction sensor in the compensated regime is predominantly determined by the control circuitry. Therefore, let us start with the signal flow diagram of the magnetic field direction sensor in the compensated regime shown in Figure 5.

The output of linear measurement bridges, represented by the vector **u***_B_*, is led to the compensator **C** (*s*), whose output simultaneously serves as the output of the magnetic field direction sensor in compensated mode ***u***. On the other hand, the output of the compensator **C** (*s*) closes the feedback loop through the gain matrix **P**. In practical implementations, this feedback loop is formed with two independent power amplifiers powering two orthogonally positioned coils, so that the magnetic field generated by the coils compensates for the measured magnetic field **H**.

Since the actual transfer function of the feedback loop is highly linear, the gain matrix **P** serves as a good model. Let us assume that the reference axis of measurement aligns with the actual *y*-axis of the gain matrix, and that *Px* and *Py* represent gains in the directions of the *x* and *y* axes, respectively, with *δ*_0_ being the phase shift in the axes, an error term describing the deviation from an ideal orthogonality. Then, the gain matrix can be represented as:

(53) $$P=\left[\begin{array}{cc} P_{x}\cdot \cos\delta_{0} & -P_{x}\cdot \sin\delta_{0}\\ 0 & P_{y} \end{array}\right].$$

On the other hand, without delving into the details of its structure, the compensator **C** (*s*) is designed to have first-order astatism, so that its input **u***_B_* is identically zero the steady state. Therefore, the angle *ϕ* of the magnetic field present on the magnetoresistive elements is small; thus, we have:

(54) $$\begin{array}{c} \sin\phi \approx \phi ,\\ \sin\left(3\cdot \phi \right)\approx 3\cdot \phi . \end{array}$$

By substituting Equation (54) into Equation (45), we obtain:

(55) $$\begin{array}{cc} {\hat{u}}_{B}\left(\phi \right) & =\underset{\mathrm{order}=0}{\underset{︸}{\left(1+g_{B}\right)\cdot \left(e_{B,1}-e_{B,3}+e_{B,5}-e_{B,6}+e_{0}\right)}}\\ & +\underset{\mathrm{order}=1}{\underset{︸}{\left(1+g_{B}\right)\cdot \frac{m_{E}\cdot H}{H_{0}}\cdot \left(K_{B}\cdot \Delta R+e_{B,2}-e_{B,4}-e_{B,7}+3\cdot e_{B,8}\right)\cdot \phi }}. \end{array}$$

So, in the case of compensated operating mode, the transfer function of the measurement bridge is linear, with the equivalent gain *A_B_* and offset *o_B_* of the measurement bridge model given by:

(56) $$\begin{array}{c} A_{B}=\left(1+g_{B}\right)\cdot \frac{m_{E}\cdot H}{H_{0}}\cdot \left(K_{B}\cdot \Delta R+e_{B,2}-e_{B,4}-e_{B,7}+3\cdot e_{B,8}\right),\\ o_{B}=\left(1+g_{B}\right)\cdot \left(e_{B,1}-e_{B,3}+e_{B,5}-e_{B,6}+e_{0}\right). \end{array}$$

By substituting Equation (56) into Equation (55) we arrive at the model of the measurement bridge in compensated operating mode:

(57) $${\hat{u}}_{B}\left(\phi \right)=A_{B}\cdot \phi +o_{B}.$$

In this case, it is evident that the transfer function of the measurement bridge model is linear; thus, the common transfer function for measurement bridges placed along the *x* and *y* axes is also linear:

(58) $${\hat{u}}_{B}\left(\phi \right)=A_{B}\cdot \phi +o_{B}.$$

However, when determining the sensor gain matrix **A**_B_, it is necessary to consider the angle *ϕ*_1_, which represents the actual direction of the measurement bridge located along the *y*-axis of the sensor, with respect to the adopted reference direction, as well as the phase shift *ϕ*_0_. Therefore, the sensor gains matrix **A**_B_ and the offset vector of the model **o**_B_ are given by:

(59) $$\begin{array}{cc} A_{B}=\left[\begin{array}{cc} \cos\phi_{1} & -\sin\phi_{1}\\ \sin\phi_{1} & \cos\phi_{1} \end{array}\right]\cdot \left[\begin{array}{cc} A_{x}\cdot \cos\phi_{0} & -A_{x}\cdot \sin\phi_{0}\\ 0 & A_{y} \end{array}\right], & o_{B}=\left[\begin{array}{c} o_{x}\\ o_{y} \end{array}\right] \end{array}.$$

Knowing that in the steady state the input **u***_B_* is constantly zero, the diagram from Figure 5 in steady state can be described as:

(60) $$A_{B}\cdot \left(H\left(\phi \right)-P\cdot \hat{u}\left(\phi \right)\right)+o_{B}=0.$$

Alternatively, after separating variables:

(61) $$A_{B}\cdot P\cdot \hat{u}\left(\phi \right)=A_{B}\cdot H\left(\phi \right)+o_{B}.$$

By solving Equation (61), we arrive at the model of the magnetic field direction sensor in compensated mode:

(62) $$\hat{u}\left(\phi \right)=P^{-1}\cdot H\left(\phi \right)+P^{-1}\cdot A_{B}^{-1}\cdot o_{B}.$$

The model (62) can be conveniently represented in notation analogous to (49) for the magnetic field direction sensor model in linear operating mode, or (37) for the magnetic field direction sensor model in direct operating mode:

(63) $$\hat{u}\left(\phi \right)=G\cdot \left[\begin{array}{c} \cos\phi \\ \sin\phi \end{array}\right]+o+e\left(\phi \right),$$

where the gain matrix of the model is denoted as **G**, the offset vector as **o**, and the residual error **e** (*ϕ*):

(64) $$\begin{array}{ccc} G=H\cdot P^{-1}, & o=P^{-1}\cdot A_{B}^{-1}\cdot o_{B}, & e\left(\phi \right)=0. \end{array}$$

The model of the magnetic field direction sensor in compensated operating mode is linear, described solely by the gain matrix **G** of the model and the offset vector **o** of the model. It should be emphasized that the zero residual error in the compensated operating mode follows from the assumptions adopted in the steady-state closed-loop model. In particular, the compensator is assumed to provide first-order astatism, so that the bridge input **u***_B_* is zero in steady state and the magnetoresistive elements operate in the locally linear region. Under these assumptions, the nonlinear harmonic contribution vanishes, and the sensor model is completely described by the gain matrix and the offset vector. In a practical implementation, however, finite loop gain and bandwidth, non-ideal orthogonality or cross-axis coupling of the compensation coils, actuator nonlinearities, amplifier offset and drift, saturation, and dynamic external magnetic fields may produce a non-zero residual error. Therefore, **e** (*ϕ*) identically equal to zero should be interpreted as a property of the ideal steady-state model rather than as a claim of zero error in a physical sensor.

In some implementations of such a sensor, an auxiliary magnetic field is applied to “invert” the characteristics of the magnetoresistive elements, which changes the sign of the gain matrix **G** of the model but does not affect the offset vector **o** of the model. The characteristic periodically changes the sign of the increment, so after demodulating the outputs of the measurement bridges, the measurement offset is eliminated. Model (63) would also be applicable in this case, but it would be described solely by the gain matrix **G** of the model, while the offset vector **o** of the model would be zero.

### 5.4. Universal Model of Magnetic Field Direction Sensor

By comparing the model of the magnetic field direction sensor in direct operating mode (37) with the model of the magnetic field direction sensor in linear operating mode (49), as well as the model of the magnetic field direction sensor in compensated operating mode (63), it can be observed that they differ only by the constant with the angle of the magnetic field, *ϕ*. Therefore, let *m_B_* be an auxiliary parameter of the magnetic field direction sensor, determined to be equal to *m_E_* if the direct operating mode is applied, and 1 otherwise. Now, the model of the magnetic field direction sensor can be represented independently of the operating mode as:

(65) $$\begin{array}{c} \phi^{\prime }=m_{B}\cdot \phi ,\\ \hat{u}\left(\phi^{\prime }\right)=G\cdot \left[\begin{array}{c} \cos\left(\phi^{\prime }\right)\\ \sin\left(\phi^{\prime }\right) \end{array}\right]+o+e\left(\phi^{\prime }\right). \end{array}$$

The measurement result, or approximate direction of the magnetic field, could be directly determined as the argument of the vector **û**. However, it should be noted that such a naive approximation would introduce unacceptably large errors, so it is necessary to transform the model (65) into a form suitable for pseudo-inversion for practical applications. Another drawback of the model (65) is that the form of the residual error (39) and (51) is not informative, as it only directly indicates individual errors in converting the components of the vector **û**, while the result is contained only in its argument. Therefore, it is suitable to replace the residual error of the measuring bridges **e** (*ϕ*′) in the model of the magnetic field direction sensor with the residual error of the magnetic field angle $\hat{e}$ (*ϕ*′), as follows:

(66) $$\begin{array}{c} \phi^{\prime }=m_{B}\cdot \phi ,\\ \hat{u}\left(\phi^{\prime }\right)=G\cdot \left[\begin{array}{c} \cos\left(\phi^{\prime }+m_{B}\cdot \hat{e}\left(\phi^{\prime }\right)\right)\\ \sin\left(\phi^{\prime }+m_{B}\cdot \hat{e}\left(\phi^{\prime }\right)\right) \end{array}\right]+o. \end{array}$$

Equating models (65) and (66) leads to an implicit expression for the residual magnetic-field angle error. The detailed derivation and its linearization around the nominal operating point are provided in Appendix A.3. The resulting approximate expression for the residual magnetic-field angle error is:

(67) $$\hat{e}\left(\phi^{\prime }\right)\approx m_{B}^{-1}\cdot {\left[\begin{array}{c} -\sin\left(\phi^{\prime }\right)\\ \cos\left(\phi^{\prime }\right) \end{array}\right]}^{T}\cdot G^{-1}\cdot e\left(\phi^{\prime }\right).$$

Considering that the gain matrix of the model **G** is constant in all considered cases, and that the residual error of the measuring bridges **e** (*ϕ*′) is a harmonic function of at most the third order, the model of the residual error of the magnetic field angle (2) is a harmonic function of at most the fourth order.

## 6. Angle Position Sensor Model

**Figure 6:**
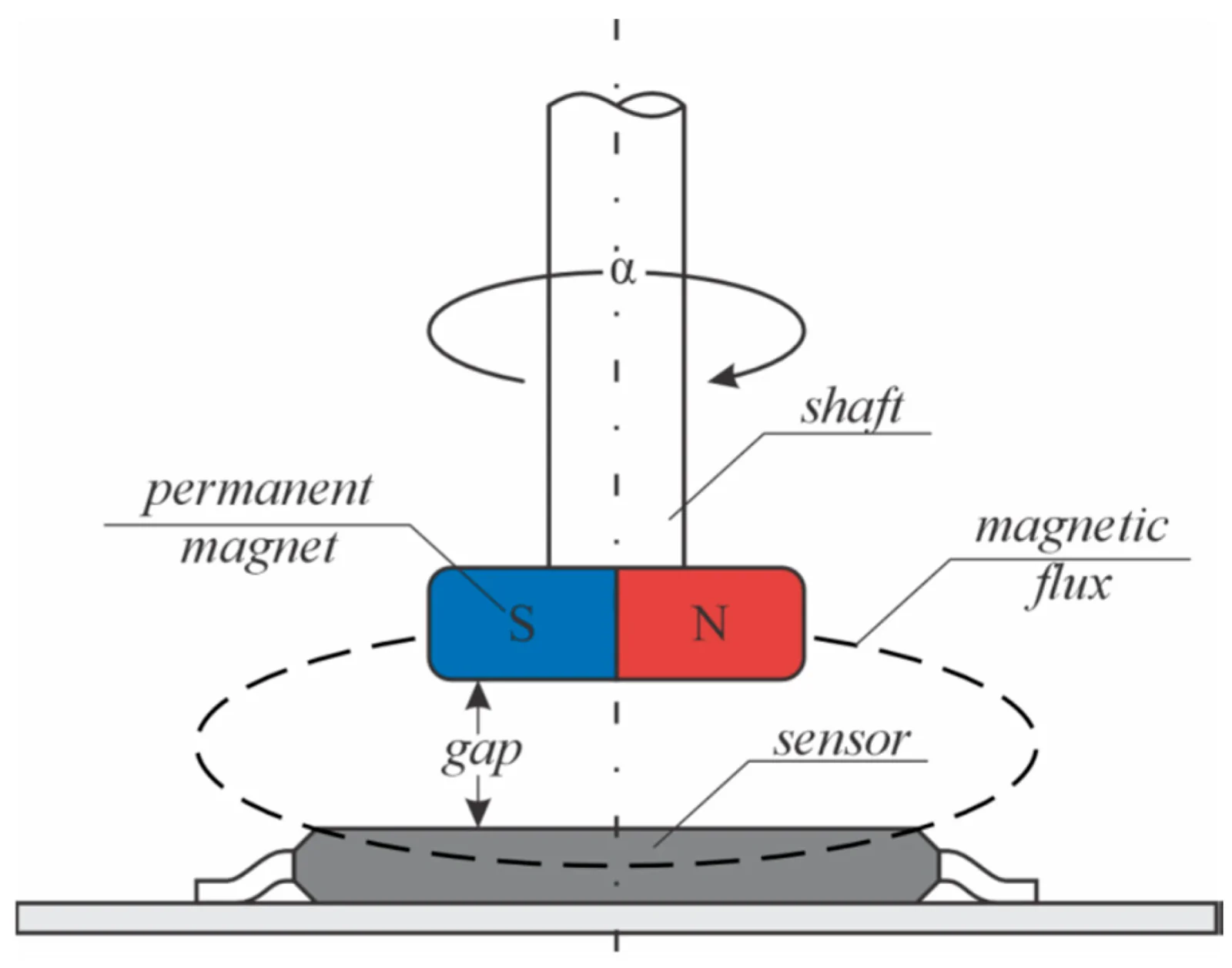
Magnetoresistive angular position transducer.

The magnetoresistive angular position transducer is depicted in Figure 6. A permanent magnet is positioned above the magnetoresistive sensor for magnetic field direction. Simultaneously, the permanent magnet is connected to the shaft whose angular position is measured, so that its magnetic field rotates with the shaft. The relationship between the shaft angle α and the magnetic field angle ϕ is equal to the number of pairs of poles of the permanent magnet, p.

Therefore, considering the sensor model for magnetic field direction depending on the operating mode (29), (42), and (52), the general form of the transfer function of the magnetoresistive angular position transducer can be described as:

(68) $$\begin{array}{l} u_{x}\left(\alpha \right)=U\cdot \cos\left(m\cdot \alpha \right),\\ u_{y}\left(\alpha \right)=U\cdot \sin\left(m\cdot \alpha \right). \end{array}$$

In Table 6, an overview of parameters *U* and *m* for the model (68) is provided for common technologies used in the fabrication of magnetoresistive angular position sensors. *U* represents the amplitude of the orthogonal sensor output signals, while *m* is the angular multiplication factor relating the mechanical shaft angle α to the angular argument of the sensor outputs. Their mode-dependent expressions follow from the corresponding magnetic field direction sensor models in (29), (42), and (52).

By observing the working principle of the magnetoresistive angle position sensor, three main causes of conversion errors can be identified: errors in converting mechanical angle into magnetic field direction *e_H_* (*α*), errors caused by external magnetic field disturbances *e_D_*, and errors in converting the magnetoresistive sensor’s magnetic field direction (67).

The results presented in [7,8,9] indicate that the nonlinear measurement error, arising from the characteristics of permanent magnets and mechanical tolerances of the system, is a slowly varying periodic function of the angular position. Therefore, a harmonic functional model of the conversion error from mechanical angle to magnetic field direction is chosen, with parameters *e_H_*_0_, *e_Ha,k_* and *e_Hb,k_*:

(69) $$e_{H}\left(\alpha \right)=e_{H0}+\sum_{k=1}^{n}\left[e_{\mathrm{Ha},k}\cdot \cos\left(k\cdot \alpha \right)+e_{\mathrm{Hb},k}\cdot \sin\left(k\cdot \alpha \right)\right].$$

On the contrary, the analysis of the influence of slowly varying external magnetic fields on magnetoresistive magnetic field direction sensors [7] indicates that it manifests as an offset error, inherently indistinguishable from the offset error of the analog interface. Therefore, in the model, it is sufficient to increase the estimated offset error of the analog interface *e*_0_ from (22), or **o** from (66), to include the effects of the external magnetic field.

Taking all of the above into consideration, and by substituting values from Table 6 into the model (66), we arrive at the model of a real angle position sensor in the form:

(70) $$\begin{array}{c} \phi^{\prime }=m\cdot \left(\alpha +e_{H}\left(\alpha \right)\right),\\ \hat{u}\left(\phi^{\prime }\right)=G\cdot \left[\begin{array}{c} \cos\left(m\cdot \left(\alpha +e_{H}\left(\alpha \right)\right)+m\cdot \hat{e}\left(m\cdot \left(\alpha +e_{H}\left(\alpha \right)\right)\right)\right)\\ \sin\left(m\cdot \left(\alpha +e_{H}\left(\alpha \right)\right)+m\cdot \hat{e}\left(m\cdot \left(\alpha +e_{H}\left(\alpha \right)\right)\right)\right) \end{array}\right]+o. \end{array}$$

However, since both the conversion error from mechanical angle to magnetic field direction *e_H_* (*α*) and the residual error of the magnetic field angle $\hat{e}$ (*ϕ*′) are slowly varying functions of small amplitudes, the effects of the second order, such that the following equality holds:

(71) $$\hat{e}\left(m\cdot \left(\alpha +e_{H}\left(\alpha \right)\right)\right)\approx \hat{e}\left(m\cdot \left(\alpha +e_{H0}\right)\right).$$

By substituting (A23) into (66), we arrive at the approximate model of the real angular position sensor in the following form:

(72) $$\hat{u}\left(\phi^{\prime }\right)=G\cdot \left[\begin{array}{c} \cos\left(m\cdot \left(\alpha +e_{H}\left(\alpha \right)+\hat{e}\left(m\cdot \left(\alpha +e_{H0}\right)\right)\right)\right)\\ \sin\left(m\cdot \left(\alpha +e_{H}\left(\alpha \right)+\hat{e}\left(m\cdot \left(\alpha +e_{H0}\right)\right)\right)\right) \end{array}\right]+o.$$

Model (72) indicates that the angle position sensor can be represented as three independent sequential segments. Notice that the central segment (72) consists of trigonometric functions with identical arguments, in which a circular modulator with an integer multiplier *m* is recognized. Therefore, let *α_H_* be the auxiliary angle, the input signal of the circular modulator represented in the mechanical coordinate system, which is determined by the nonlinear harmonic segment given by:

(73) $$\alpha_{H}=\alpha +e_{H}\left(\alpha \right)+\hat{e}\left(m\cdot \alpha +m\cdot e_{H0}\right),$$

Then, the output of the circular modulator **û*_M_*** is given by:

(74) $${\hat{u}}_{M}\left(\alpha \right)=\left[\begin{array}{c} \cos\left(m\cdot \alpha_{H}\right)\\ \sin\left(m\cdot \alpha_{H}\right) \end{array}\right].$$

By substituting (74) into (72), we obtain the remaining linear segment of the model in the following form:

(75) $$\hat{u}\left(\alpha \right)=G\cdot {\hat{u}}_{M}\left(\alpha \right)+o.$$

Separating the linear and nonlinear segments of the model significantly facilitates practical implementation and suggests a method for measurement compensation.

An important property of the nonlinear harmonic segment defined in (73) is the strictly limited harmonic content of the residual magnetic field angle error $\hat{e}$. As follows from (67), this residual error is confined to low-order harmonics, with the fourth harmonic being the highest possible order. More specifically, depending on the operating mode, power source, and presence of a parasitic AMR element, the residual error is either zero or contains only third- or fourth-order harmonic components, as summarized in Table 7.

For the compensated operating mode, the zero residual error refers to the ideal steady-state closed-loop model defined in Section 5.3. Practical non-idealities of the feedback path may introduce additional residual components not included in the present model. Furthermore, the residual magnetic field angle error $\hat{e}$ (*ϕ*′) does not contain a constant component in any of the considered cases, which follows from the adopted reference position. This is a direct consequence of the harmonic structure of the measuring-bridge residual errors in (39), (51), and (64), in which the first harmonic does not participate.

**Figure 7:**
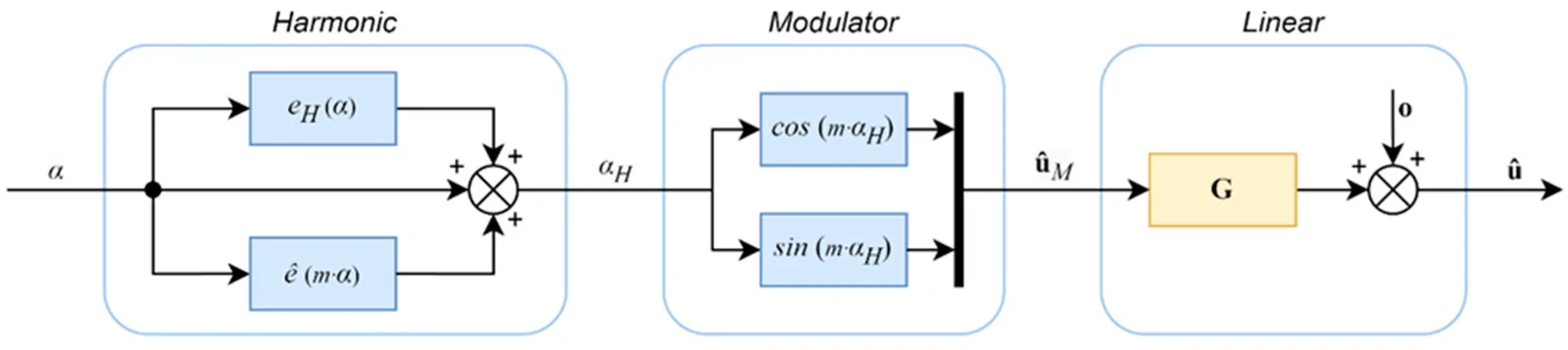
Signal flow diagram of the angle position sensor model.

Signal flow diagram of the magnetoresistive angular position transducer model, where the linear segment, circular modulator, and harmonic nonlinear segment are explicitly separated, is depicted in Figure 7.

The proposed decomposition differs from conventional angular-error descriptions in that the dominant linear imperfections and the nonlinear periodic error are represented in separate functional blocks. The linear segment contains gain imbalance, offset, phase-shift, and other affine imperfections of the two-channel sensor output, while the circular modulator represents the ideal angular transformation between the mechanical and electrical coordinate systems. The remaining nonlinear contribution is confined to the harmonic segment, whose order is explicitly linked to the sensor technology, bridge excitation, operating mode, and parasitic AMR contribution.

This separation has two practical advantages. First, linear imperfections can be identified and compensated independently before angular demodulation. Second, the remaining angular error can be represented by a low-order harmonic model, enabling a reduced number of calibration parameters compared with a general nonlinear mapping. In contrast to models focused on individual physical error sources [7,8,9], the proposed representation therefore provides a common signal-flow framework suitable for different AMR, GMR, and TMR sensor configurations.

One of the drawbacks of model (73) is the practical difficulties due to limitations in the application of the functional model of the error in converting mechanical angle to magnetic field direction (69). Namely, it is a harmonic model based on the mechanical angle *α*, which is generally known only through its product with a constant *m*. Reconstructing the original angle is not always possible, especially if an external reference position does not exist. In such a situation, it is necessary to apply a reduced model of the conversion error from mechanical angle to magnetic field direction, in which parameters *e_Ha,k_* and *e_Hb,k_* can be non-zero only for *k* divisible by *m*.

## 7. Model Verification

This section systematically addresses the key elements related to the experimental validation of the magnetoresistive sensor model for angular position measurement, including the experimental setup, configurations, and model verification.

### 7.1. Experimental Verification for AMR Sensor

**Figure 8:**
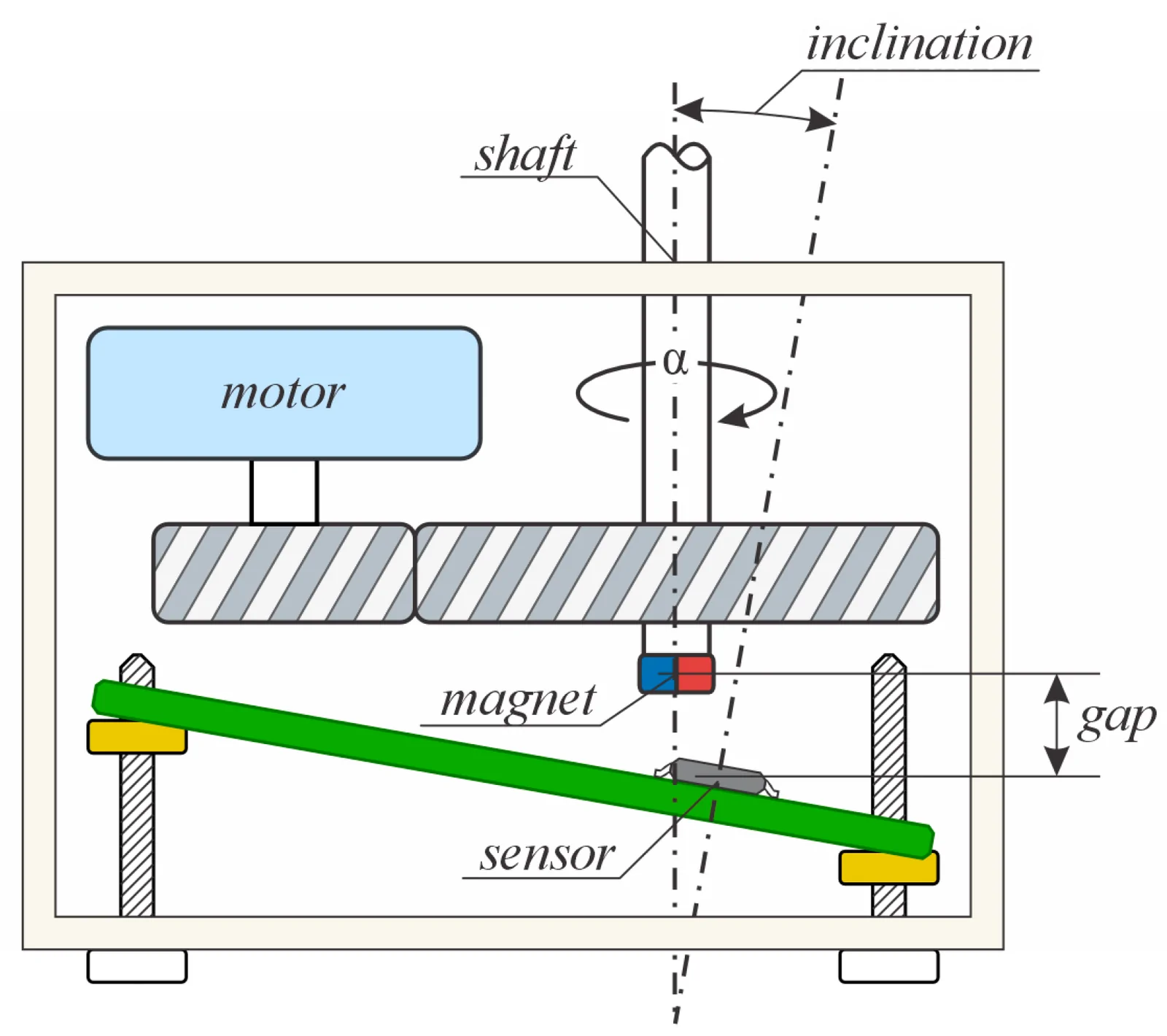
Block diagram of the experimental setup.

The experimental apparatus used for the verification of the magnetoresistive angular position sensor model was developed with the aim of providing precise data for analysis in multiple independent configurations, as shown in Figure 8.

A low-power two-phase motor is connected to the working shaft via a transmission system. At the end of the working shaft, a permanent SmCo magnet with a single pole pair and a nominal magnetic induction of 200 mT is mounted. The end of the working shaft with the permanent magnet is positioned above the AMR angular position sensor in direct operating mode, at a relatively large distance of approximately d ≈ 2 mm, such that the magnetic field intensity is near the lower boundary of the selected sensor’s operating range. The magnetoresistive sensor itself is mounted on a printed circuit board (PCB) that contains the electronic subsystem of the apparatus. The PCB can be positioned at an adjustable angle relative to the axis of the magnet, enabling verification of the system’s behavior even in cases where the sensor and magnet are not perfectly aligned.

All experiments were conducted under controlled conditions to eliminate external influences such as electromagnetic interference. The experiments were conducted in three basic configurations, as presented in Table 8.

In experimental configuration (I), the axes of the magnet and sensor were “almost” aligned, allowing this configuration to be considered free of sensor inclination and external magnetic interference. In the second experimental configuration (II), a constant magnetic interference field was introduced by carefully positioning additional permanent magnets in close proximity to the sensor. Unfortunately, this also required the axes of the magnet and sensor to be translationally offset relative to each other. Finally, in the third experimental configuration (III), the largest possible angle between the magnet and sensor axes was chosen, where the magnetic field intensity in the sensor plane remained within the sensor’s nominal operating range. In this configuration, the inclination of the sensor’s printed circuit board was approximately 15°. The experimental design is robust, as it includes three configurations that account for varying conditions. This variation enables a comprehensive analysis of sensor performance under different operating conditions.

The verification of the magnetoresistive angular position sensor model was conducted in three independent experiments, one for each experimental configuration. The experiments began by setting the maximum allowed angular velocity of the motor. Once the experimental setup reached a steady state during at least one full rotation of the working shaft, the sensor signals were recorded.

The recorded signals were processed using a set of automated scripts. During execution, signals resulting from the A/D conversion of the measurement bridge signals were extracted, and the residual offset of the analog interface was removed. To reduce the influence of noise on the process of identifying the parameters of the magnetoresistive angular position sensor model, the sample was formed as the average value of all recorded full rotations of the shaft. Then, the parameters of the sensor model were identified by applying the methods described in [32] to the formed sample. The harmonic error coefficients were identified up to the tenth order of the angle in the electrical coordinate system, and up to the twentieth order in the mechanical coordinate system. No a priori truncation of the harmonic order was imposed. The identification was intentionally extended to substantially higher orders to quantify possible unmodelled harmonic components and to assess the model order required by the experimental data.

**Figure 9:**
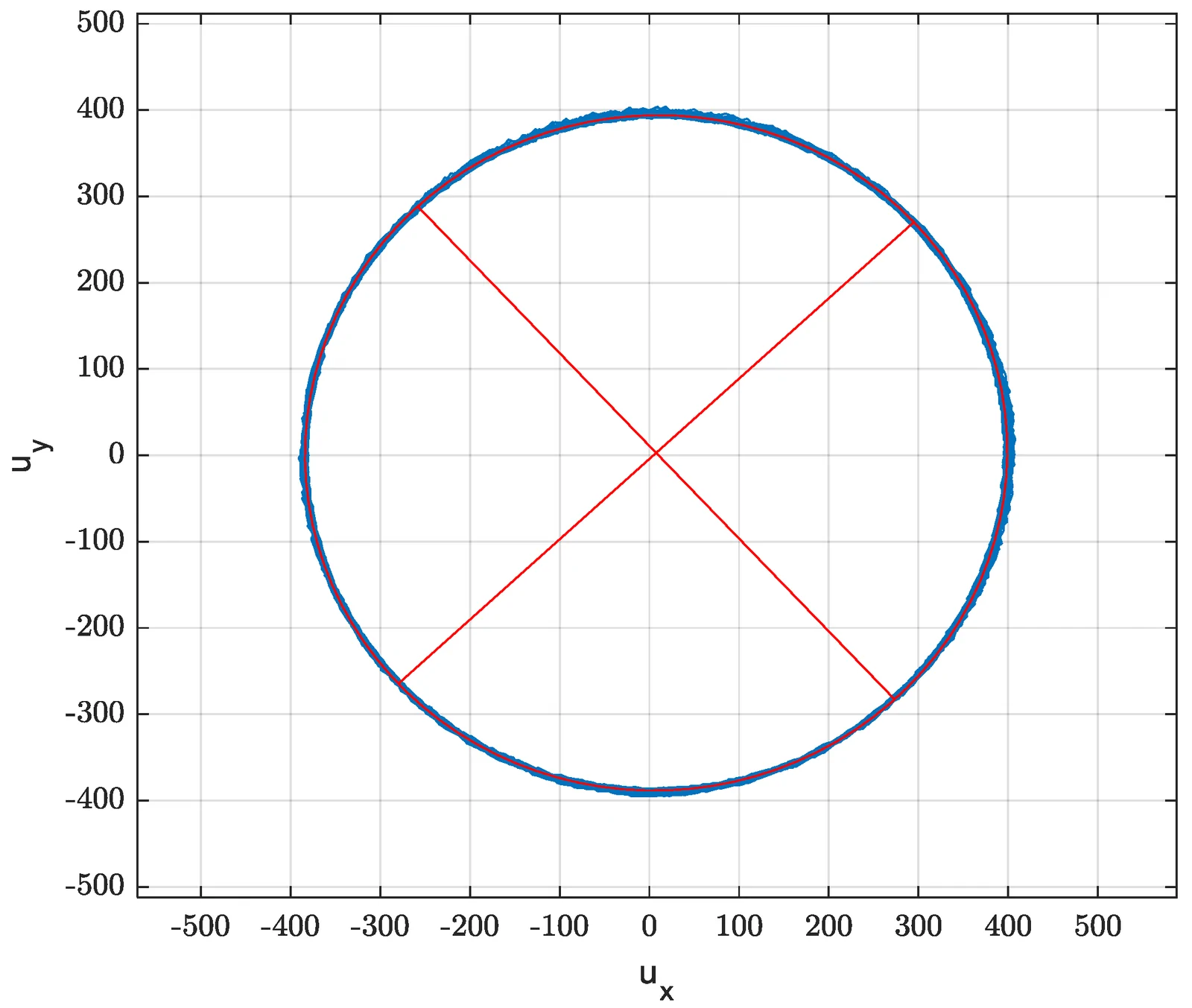
Input vector trace (blue) and identified linear segment of the model (red), experimental configuration I.

Since the equipment does not have an external reference position indicator, it is, according to the discussion in Section 5.4 and Section 6 regarding the application of AMR sensors in direct operating mode, impossible to identify odd harmonics of the angle in the mechanical coordinate system. Figure 9 shows the input signal trace recorded during the experiment conducted with experimental configuration I.

In all the conducted experiments, the input signal trace and the identified linear segment of the model show excellent agreement with the model (73), (74), and (75), clearly supporting the hypothesis of separating the linear segment of the model, the circular modulator, and the nonlinear harmonic segment of the model presented in Section 6. Indirectly, this result also confirms the validity of the independent linear compensation of measurements before demodulation, applied in the sensor model, as described in Section 5.3 and Section 6.

**Figure 10:**
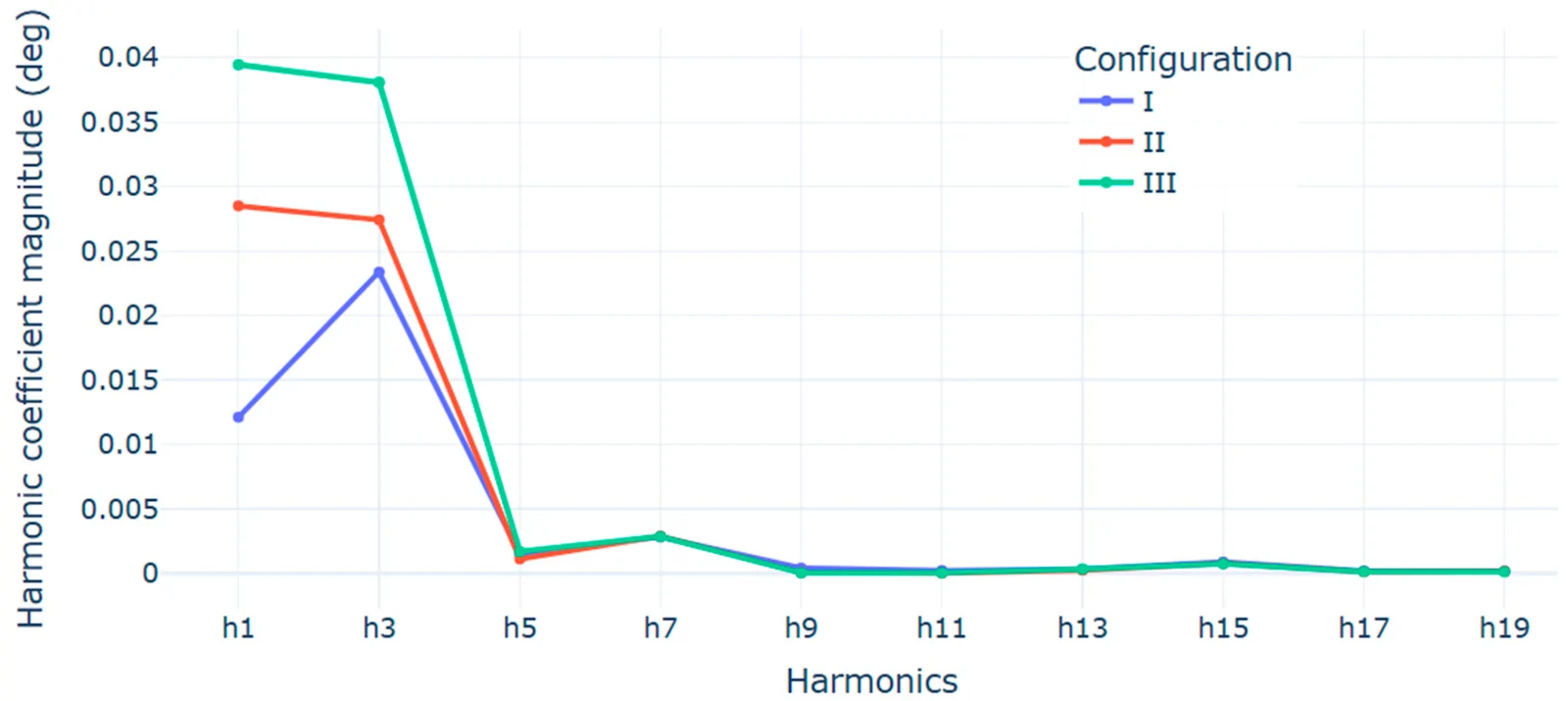
Magnitudes of the harmonic segment coefficients of the model.

The magnitudes of the identified harmonic segment coefficients of the model depending on the harmonic order are shown in Figure 10.

Figure 10 indicates that the identified harmonic spectrum is dominated by low-order components, while higher-order terms have progressively smaller amplitudes. To quantify their relative significance across the three experimental configurations, the magnitudes of the higher-order harmonic coefficients were normalized to the dominant harmonic coefficient in each configuration. The resulting relative magnitudes are summarized in Table 9.

The obtained experimental results confirm that the harmonic error spectrum is dominated by low-order components. As summarized in Table 9, the third-order coefficient amounts to 6.15%, 3.90%, and 4.25% of the dominant harmonic coefficient for configurations I, II, and III, respectively. The fourth-order contribution is somewhat larger, reaching 12.03%, 10.11%, and 7.22%, respectively. From the fifth order onward, the relative coefficients decrease substantially; with the exception of the eighth-order component, all coefficients from the fifth to the eighth order remain below 1.73% of the dominant component. Furthermore, all identified coefficients above the eighth order are below 0.81% of the dominant harmonic coefficient. The relatively small third-order contribution is consistent with partial compensation of the corresponding error terms due to correlated manufacturing deviations of the magnetoresistive elements within the same measurement bridge. For the geometry and manufacturing process of the selected AMR sensor [33], such correlation reduces the contributions associated with the harmonic functions*f*_6_ and *f*_7_, as indicated by Table A1. The non-zero fourth- and eighth-order components indicate that the experimentally observed harmonic structure is not strictly limited to the lowest model orders; however, their practical significance must be evaluated through their effect on the prediction accuracy of the identified model.

**Figure 11:**
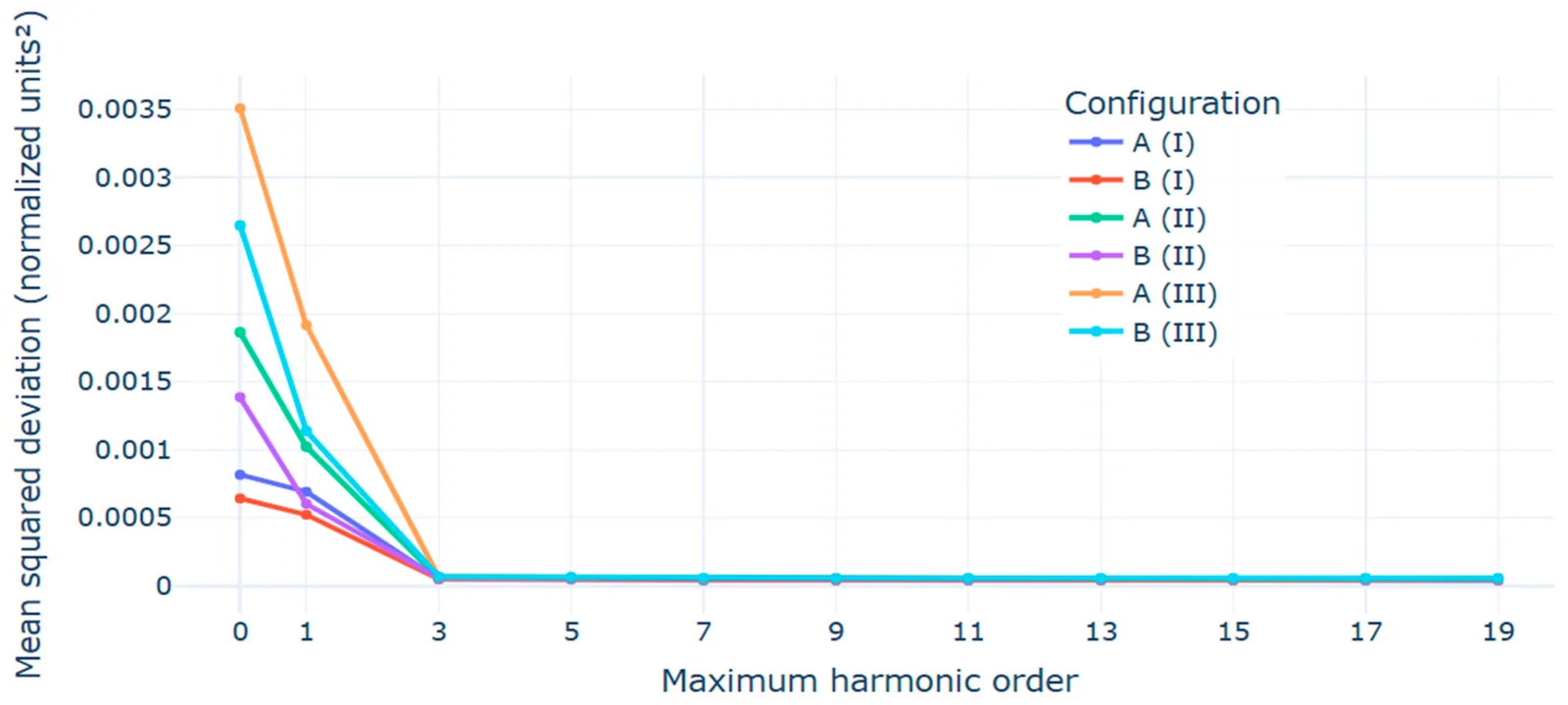
Mean squared deviation between measured and predicted normalized signals of bridges A and B as a function of the harmonic order of the model.

To assess the practical significance of the identified higher-order harmonics, models with progressively increasing harmonic order were used as predictors of the measured bridge signals. The mean squared deviation between the predicted and measured normalized bridge signals was then calculated as a function of the retained harmonic order. The results are shown in Figure 11. The results of this analysis are shown in Figure 11.

Figure 11 shows that the mean squared deviation decreases rapidly with increasing harmonic order. The dominant improvement is obtained by including the low-order harmonic terms, while for model orders of four and above the mean squared deviation remains on the order of 5·10^−5^, with only minor differences between the three experimental configurations. Since the bridge signals are expressed in normalized units, the MSE is given in squared normalized units. This residual level is comparable to the measurement-noise floor of the bridge-voltage acquisition system, indicating that further increases in harmonic order do not provide a practically meaningful improvement in prediction accuracy.

It should therefore be emphasized that the reduced harmonic representation was not imposed a priori. Harmonic coefficients were identified to substantially higher orders, and the effective model order was assessed from both the identified coefficient magnitudes and the resulting prediction error. Although measurable third- and fourth-order components are present, the contribution of still higher orders to the prediction accuracy becomes comparable to the measurement noise. Thus, for the investigated operating conditions, a low-order harmonic model provides an adequate reduced representation of the measured sensor behavior.

This conclusion applies to the investigated configurations and should not be interpreted as a universal truncation rule. The analytical derivation relies on small-error approximations, and sufficiently large mechanical misalignment, strongly spatially non-uniform magnetic disturbances, or other conditions outside these assumptions may increase the significance of higher-order components. In such cases, the appropriate harmonic order should be determined from the measured data rather than prescribed in advance.

The experimental results indicate that, for the investigated configurations, the contribution of the mechanical-angle-to-magnetic-field conversion error *e_H_* (*α*) did not produce substantial additional high-order harmonic content. This observation is consistent with the low-order representation obtained for the tested magnet geometry and operating conditions. However, since the complete model relies on small-error approximations, this result should not be generalized to substantially larger mechanical misalignment or strongly spatially non-uniform magnetic disturbances.

### 7.2. Simulation-Based Verification for GMR and TMR Sensors

Numerical case studies were performed to examine the applicability of the proposed formulation to GMR and TMR sensors beyond the experimentally investigated AMR device. Each simulated sensor comprised two orthogonal full bridges with four active elements and operated in the direct mode. A uniform magnetic field sufficiently strong to align the free-layer magnetization with the field direction was assumed. With one pole pair and unity electrical-to-mechanical angular scaling, the mechanical, field-direction, and magnetization angles coincided. This setup isolates the element and bridge contributions from errors associated with the permanent magnet and its mechanical assembly.

The simulation parameters are summarized in Table 10. Both technologies were described using the common aggregate element model, with their different MR values entering through this amplitude ratio.

Geometric, composition, and reference-axis deviations were represented by independent zero-mean Gaussian variables. The geometric deviation affected both R and ΔR, thereby correlating their aggregate deviations within each element. When enabled, the parasitic AMR coefficient was normally distributed with mean 0.05∙ΔR and relative standard deviation of 10%. These values are declared simulation assumptions. In particular, the 5% AMR contribution is an adjustable coefficient of the second-harmonic resistance term, not a universal property of GMR or TMR devices. Cases with this coefficient set to zero provide controlled limiting cases. Eight configurations were evaluated by combining the two technologies, CV/CC excitation, and AMR on/off.

Two calculation paths were compared. The nonlinear numerical path directly evaluated the aggregate resistance model, the resistive-network equations, and angular demodulation. Linear parameters were identified using 1440 uniformly spaced sample points over one full rotation and evaluated on a disjoint angular grid shifted by half a sample interval. The analytical path propagated the element deviations through the nominal bridge Jacobian and the angular projection, consistently retaining terms of first order in the parameter deviations. Its constant and fundamental components were separated using the analytical signal itself. The analytical prediction was therefore derived from the element parameters, without fitting the nonlinear numerical angular residual.

**Figure 12:**
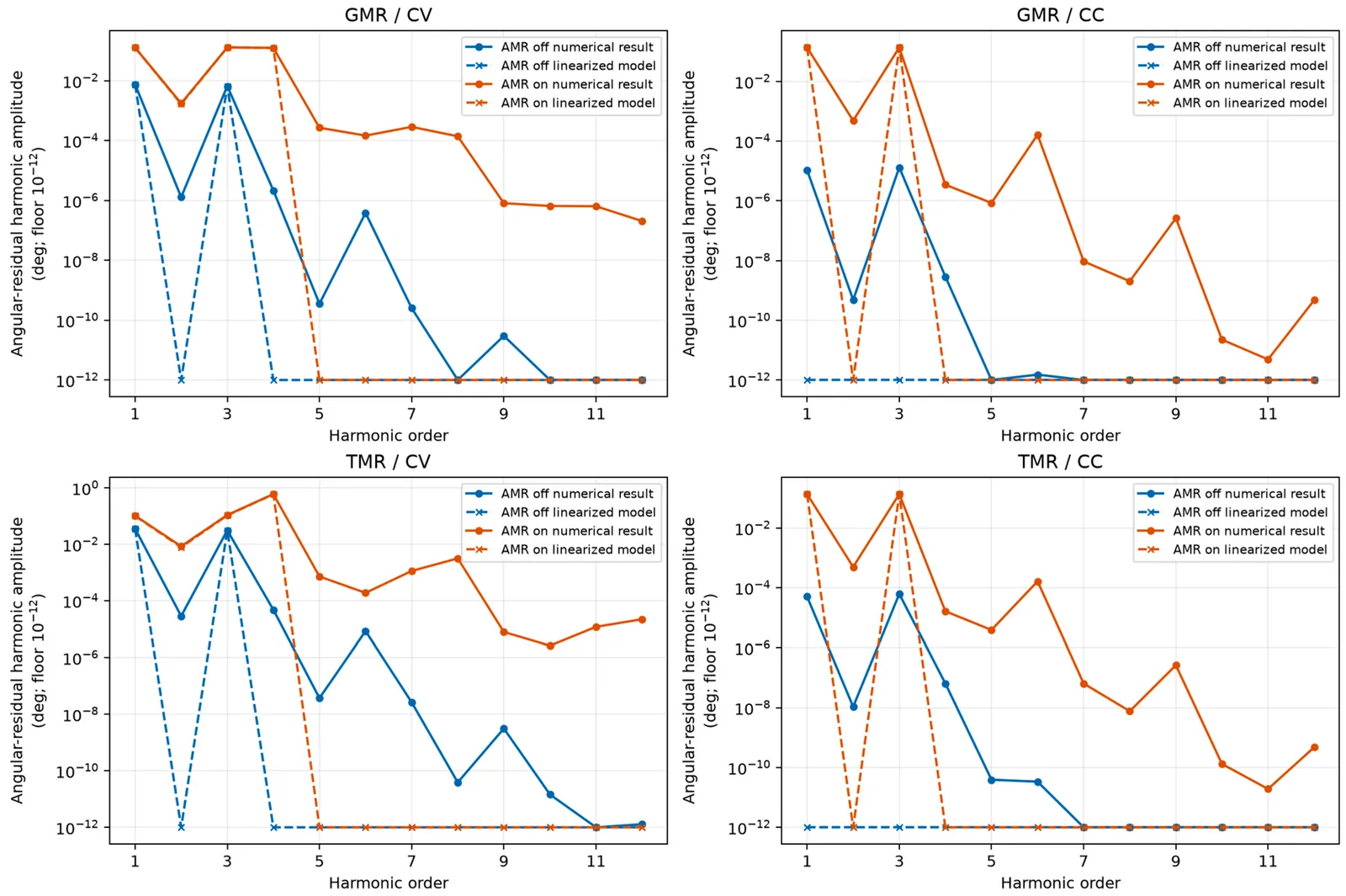
Harmonic amplitudes of the angular residual for the representative GMR and TMR realizations.

Figure 12 compares the angular-residual spectra of a representative individual realization for each configuration. Fourier analysis was extended through the twelfth order without truncating the numerical signal at the analytical bound. The linearized model reproduced the predicted maximum orders: four for CV with parasitic AMR, three for CV without parasitic AMR and CC with parasitic AMR, and zero residual for CC without parasitic AMR. These bounds apply only to the linearization in parameter deviations, while the nonlinear numerical calculation retains smaller higher-order terms. In particular, the numerical CC/off residual is nonzero, even though its analytical first-order prediction vanishes.

**Figure 13:**
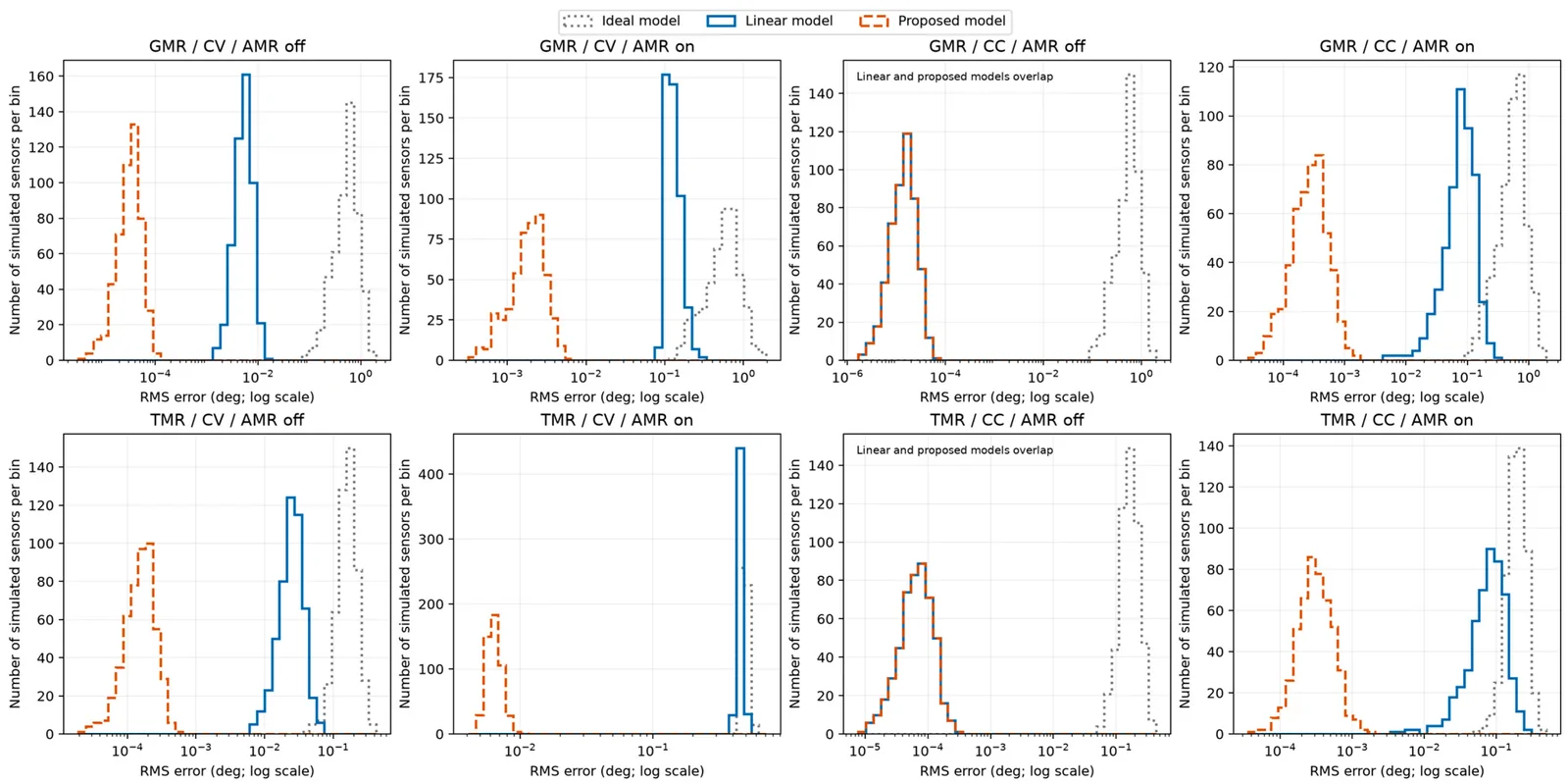
Monte Carlo distributions of RMS angular errors for the Ideal, Affine, and Proposed models. The three histograms use common logarithmic bins within each panel.

The dependence on manufacturing variability was evaluated using 500 Monte Carlo realizations for each configuration, with the same normalized random deviations reused across configurations for paired comparisons. Three levels of model description were assessed in the angular domain. The Ideal model uses direct demodulation, without correction. The Linear model accounts for offset, gain imbalance, and nonorthogonality before demodulation. Finally, the Proposed model accounts for the analytical harmonic prediction. The error of each model is the difference between the resulting numerical angular residual and its prediction. Figure 13 shows the resulting distributions of each error RMS over a complete rotation, computed separately for every realization.

Table 11 reports the means and 95th percentiles of the resulting distributions of each error RMS.

The linear model removes much of the error associated with offsets, gain imbalance, and nonorthogonality, while the proposed harmonic model accounts for the remaining systematic angular dependence. For CV excitation with parasitic AMR, the mean RMS error decreased from 0.130° for the Linear model to an approximation error of 0.002° for the Proposed model in the GMR case, and from 0.450° to 0.006° in the TMR case. The corresponding 95th-percentile approximation errors were 0.0037° and 0.0079°. Under CC excitation without parasitic AMR, the Linear and Proposed model distributions overlap because the analytical harmonic residual is zero. These comparisons characterize the selected parameter distributions and do not establish a universal ranking of the two technologies.

**Figure 14:**
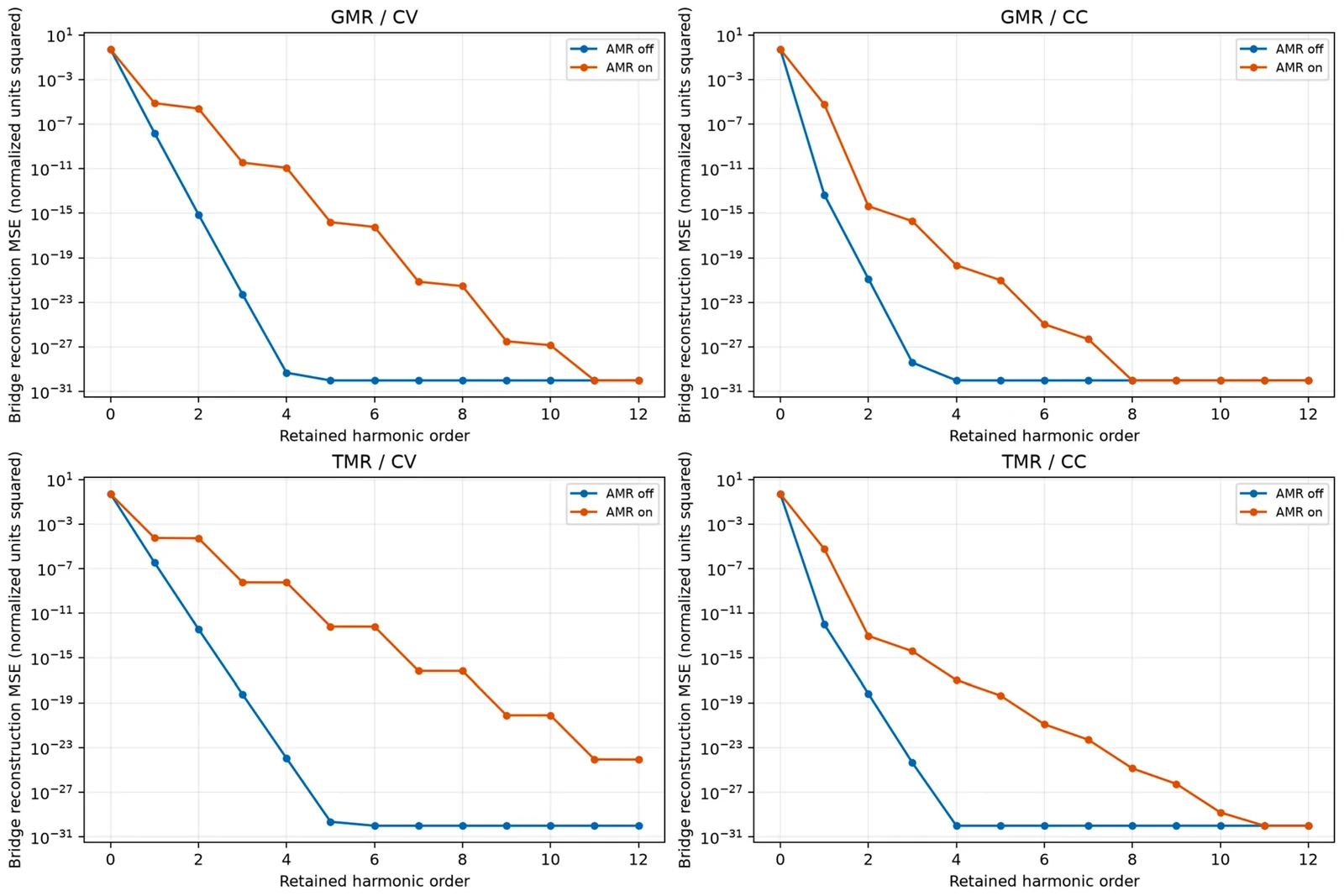
Mean squared reconstruction error of the normalized bridge signals as a function of retained harmonic order.

Figure 14 provides a complementary assessment of harmonic truncation at the bridge-signal level.

The reconstruction error decreases rapidly as the dominant harmonics are included. Among the eight representative cases, the largest MSE at order four was (5.85∙10^−9^), obtained for TMR/CV with parasitic AMR. These results describe reconstruction of noiseless synthetic signals and do not establish an experimental noise floor or sensor accuracy. Small higher-order components in the nonlinear calculation remain compatible with a model derived by neglecting higher-order products of parameter deviations.

Together, the spectral comparison, Monte Carlo statistics, and reconstruction analysis support the numerical consistency of the common formulation for the investigated direct-mode GMR/TMR configurations.

## 8. Conclusions

This study presented a parametric formalism that provides a systematic framework for constructing unified models of magnetoresistive angular position sensors based on AMR, GMR, and TMR sensing elements. The model was developed from the physical behavior of the magnetoresistive elements, extended to realistic bridge configurations with non-ideal excitation and analog interface effects, and then incorporated into a complete angular position sensor model that accounts for realistic magnetic-field conversion, manufacturing tolerances, and geometric misalignment between the sensor, magnet, and rotation axis.

The derived model shows that the behavior of a magnetoresistive angular position sensor can be represented by a functional sequence of a linear segment, a circular modulator, and a nonlinear harmonic segment. A key contribution of the proposed formalism is the explicit separation of linear imperfections, angular modulation, and nonlinear harmonic error into functionally distinct model segments. This decomposition preserves the connection between the physical error sources and the resulting sensor-level angular error, while enabling independent identification and compensation of the linear imperfections before angular demodulation. At the same time, the remaining nonlinear contribution is reduced to a low-order harmonic representation, which facilitates model identification, calibration, and compensation. The resulting formulation is therefore suitable for analytical error evaluation, tolerance analysis, Monte Carlo analysis, calibration procedure development, and compensation algorithm design.

The proposed model was experimentally verified using an AMR-based angular position sensor across three distinct configurations. The experimental results showed good agreement between the theoretical predictions and the measured sensor behavior. In particular, the identified harmonic coefficients confirmed that the dominant residual error can be accurately represented by low-order harmonic terms, with a reduced low-order model providing an accurate approximation of the tested AMR configurations. These results also confirmed the validity of separating the sensor model into a linear segment, circular modulator, and a nonlinear harmonic segment.

To complement the experimental verification performed with the AMR sensor, the applicability of the proposed formulation to GMR- and TMR-based sensors was examined through numerical case studies covering constant-voltage and constant-current excitation, with and without a parasitic AMR contribution. The nonlinear numerical simulations reproduced the low-order harmonic structure predicted by the analytical model, while Monte Carlo analysis confirmed that the proposed harmonic correction substantially reduces the model error. These results support the numerical consistency of the unified formulation across the investigated AMR, GMR, and TMR sensor technologies, while experimental verification with physical GMR and TMR devices remains necessary.

Future work should extend experimental validation to GMR- and TMR-based angular sensors, investigate different magnet geometries and stronger external interference conditions, and implement real-time model-based measurement in practical embedded systems.

## Figures and Tables

**Table 1:** Parameters of the integrated magnetoresistive element model.

Technology	*R*	Δ*R*	*θ_E_*	*m_E_*
AMR	$R_{0}+\frac{\Delta R_{A}}{2}$	$\frac{\Delta R_{A}}{2}$	0°	2
Linear AMR	$R_{0}+\frac{\Delta R_{A}}{2}$	$\frac{\Delta R_{A}}{2}$	90°	2
GMR/TMR	*R*_0_	Δ*R_G_*	0°	1
Linear GMR/TMR	*R*_0_	Δ*R_G_*	90°	1

**Table 2:** Nominal resistances of bridge circuit elements.

Element	One Active	Two Active	Four Active
*R*_1_	*R*	*R* + Δ*R_E_*(*θ*′)	*R* + Δ*R_E_*(*θ*′)
*R*_2_	*R*	*R*	*R* − Δ*R_E_*(*θ*′)
*R*_3_	*R*	*R*	*R* − Δ*R_E_*(*θ*′)
*R*_4_	*R* + Δ*R_E_*(*θ*′)	*R* + Δ*R_E_*(*θ*′)	*R* + Δ*R_E_*(*θ*′)

**Table 3:** The nominal amplification of the magnetoresistive measuring bridge depends on the configuration.

Configuration	CV, 4 Active	CC, 2 Active	CC, 4 Active
*K_B_*	$\frac{A\cdot V_{0}}{R}$	$\frac{A\cdot I_{0}}{2}$	*A* ⋅ *I*_0_

**Table 4:** The parameters of the measurement bridge model depend on the configuration.

Elements No	Source	*K_B_*	*k*_1_	*k*_2_
4	CV	$\frac{A\cdot V_{0}}{R}$	1	2
2	CC	$\frac{A\cdot I_{0}}{2}$	$\frac{1}{4}$	0
4	CC	*A* ⋅ *I*_0_	$\frac{1}{4}$	0

**Table 5:** The harmonic functions *fi* for the application of linear magnetoresistive elements.

*i*	*f_i_*(*θ*′)	*f_i_*(*θ′,θ_E_* = 90°)	*f_i_*(*ϕ*)
1	0	1	1
2	cos(*θ*′ − *θ_E_*)	sin*θ*′	$\approx \frac{m_{E}\cdot H}{H_{0}}\cdot \sin\phi$
3	sin(*θ*′ − *θ_E_*)	−cos*θ*′	≈−1
4	cos(*θ*′ + *θ_E_*)	−sin*θ*′	$\approx -\frac{m_{E}\cdot H}{H_{0}}\cdot \sin\phi$
5	cos(2 ⋅ *θ*′)	cos(2 ⋅ *θ*′)	≈1
6	cos(2 ⋅ *θ*′ − 2 ⋅ *θ_E_*)	−cos(2 ⋅ *θ*′)	≈−1
7	sin(2 ⋅ *θ*′ − 2 ⋅ *θ_E_*)	−sin(2 ⋅ *θ*′)	$\approx -2\cdot \frac{m_{E}\cdot H}{H_{0}}\cdot \sin\phi$
8	cos(3 ⋅ *θ*′ − *θ_E_*)	sin(3 ⋅ *θ*′)	$\begin{array}{l} 3\cdot \frac{m_{E}\cdot H}{H_{0}}\cdot \left(1-{\left(\frac{m_{E}\cdot H}{H_{0}}\right)}^{2}\right)\cdot \sin\phi \\ +{\left(\frac{m_{E}\cdot H}{H_{0}}\right)}^{3}\cdot \sin\left(3\cdot \phi \right) \end{array}$

**Table 6:** The parameters of the magnetoresistive angular position transducer model depend on the configuration.

Operating Mode	*U*	*m*
Direct AMR	*K_B_*⋅Δ*R*	2 ⋅ *p*
Direct GMR/TMR	*K_B_*⋅Δ*R*	*p*
Linear AMR	$2\cdot \frac{K_{B}\cdot \Delta R}{H_{0}}\cdot H$	*p*
Linear GMR/TMR	$\frac{K_{B}\cdot \Delta R}{H_{0}}\cdot H$	*p*
Compensated	*A* ⋅ *H*	*p*

**Table 7:** Form of the residual error of the magnetic field angle.

Parasitic AMR	Operating Mode	Power Source	$\hat{e}\left(\phi^{\prime }\right)$
Yes	Direct	CV	Harmonic 4th order
Yes	Direct	CC	Harmonic 3rd order
No	Direct	CV	Harmonic 3rd order
No	Direct	CC	=0
Yes	Linear	CV	Harmonic 4th order
Yes	Linear	CC	=0
No	Linear	-	=0
-	Compensated	-	=0

**Table 8:** Overview of experimental configurations.

Configuration	Description
I	Aligned axes of the sensor and magnet, no sensor inclination, no interfering magnetic field
II	The axes of the sensor and magnet are not aligned, no sensor inclination, constant interfering magnetic field.
III	The axes of the sensor and magnet are not aligned, significant sensor inclination, no interfering magnetic field.

**Table 9:** Higher-order harmonic coefficients normalized to the dominant harmonic coefficient.

Configuration	3rd	4th	5th	6th	7th	8th
I	6.15%	12.03%	1.73%	0.92%	1.35%	3.76%
II	3.90%	10.11%	0.18%	0.06%	0.84%	2.69%
III	4.25%	7.22%	0.09%	0.07%	0.80%	1.88%

**Table 10:** Parameters of the GMR and TMR simulations.

Parameter	GMR	TMR
Midpoint element resistance, R	1 kΩ	1 kΩ
Magnetoresistance ratio, MR	20%	150%
Fundamental amplitude ratio, ΔR/R	0.091	0.428
CV excitation/total CC current per bridge	5 V/5 mA	5 V/5 mA
Relative geometric deviation, standard deviation	0.2%	0.2%
Additional relative composition deviation, standard deviation	0.5%	0.5%
Reference-axis deviation, standard deviation	0.2°	0.2°
Mean parasitic AMR coefficient, r_4_/ΔR	0 or 5%	0 or 5%
Relative AMR-coefficient standard deviation, when enabled	10%	10%

**Table 11:** Mean RMS angular error and its 95th percentile, expressed as mean (95th percentile).

Type	Excitation	Parasitic AMR	Ideal Model	Linear Model	Proposed Model
GMR	CV	off	0.58 (1.13)	0.006 (0.009)	3.45∙10^−5^ (6.70∙10^−5^)
GMR	CV	on	0.60 (1.16)	0.130 (0.191)	200∙10^−5^ (370∙10^−5^)
GMR	CC	off	0.58 (1.14)	1.60∙10^−5^ (3.32∙10^−5^)	1.60∙10^−5^ (3.32∙10^−5^)
GMR	CC	on	0.59 (1.16)	0.086 (0.166)	32.6∙10^−5^ (75.1∙10^−5^)
TMR	CV	off	0.17 (0.26)	0.026 (0.045)	16.8∙10^−5^ (32.1∙10^−5^)
TMR	CV	on	0.48 (0.53)	0.450 (0.482)	641∙10^−5^ (791∙10^−5^)
TMR	CC	off	0.17 (0.27)	7.54∙10^−5^ (15.6∙10^−5^)	7.54∙10^−5^ (15.6∙10^−5^)
TMR	CC	on	0.19 (0.30)	0.085 (0.16)	35.7∙10^−5^ (76.2∙10^−5^)

## Data Availability

The raw data supporting the conclusions of this article will be made available by the authors on request.

## Appendix A. Detailed Derivations of the Magnetoresistive Element and Bridge Models

### Appendix A.1. Magnetoresistive Element Model Derivation

Grouping the individual manufacturing deviations into aggregate parameter errors reduces the real magnetoresistive element model to:

(A1) $${\hat{R}}_{E}\left(\theta^{\prime }\right)\approx \left(R+r_{E,1}\right)\pm \left(\Delta R+r_{E,2}\right)\cdot \cos\left(\theta^{\prime }-\theta_{E}+r_{E,3}\right)+r_{E,4}\cdot \cos\left(2\cdot \theta^{\prime }\right).$$

Let **r***_E_* denote the vector of aggregate errors of the magnetoresistive element model parameters:

(A2) $$r_{E}=\left[\begin{array}{c} r_{E,1}\\ r_{E,2}\\ r_{E,3}\\ r_{E,4} \end{array}\right]=\left[\begin{array}{c} \varepsilon_{E,1}\cdot R\\ \left(\varepsilon_{E,1}+\varepsilon_{E,2}+\varepsilon_{E,1}\cdot \varepsilon_{E,2}\right)\cdot \Delta R\\ \varepsilon_{E,3}\\ \left(1+\varepsilon_{E,1}+\varepsilon_{E,2}+\varepsilon_{E,1}\cdot \varepsilon_{E,2}\right)\cdot \varepsilon_{E,4}\cdot \Delta R \end{array}\right].$$

The conversion error of the magnetoresistive element can be approximated by applying a first-order linearization around the nominal operating point **r***_E_* = 0:

*e_E_*(*θ*′) ≈ *D***_r_***R_E_*(*θ*′) ⋅ **r***_E_*. (A3)

The gradient of this model with respect to the error parameter vector is:

(A4) $$D_{r}R_{E}\left(\theta^{\prime }\right)={\left[\begin{array}{c} 1\\ \cos\left(\theta^{\prime }-\theta_{E}+r_{E,3}\right)\\ \left(\Delta R+r_{E,2}\right)\cdot \sin\left(\theta^{\prime }-\theta_{E}+r_{E,3}\right)\\ \cos\left(2\cdot \theta^{\prime }\right) \end{array}\right]}^{T}.$$

Neglecting second-order effects and evaluating the gradient at the nominal operating point gives:

(A5) $$D_{r}R_{E}\left(\theta^{\prime }\right)={\left[\begin{array}{c} 1\\ \cos\left(\theta^{\prime }-\theta_{E}\right)\\ \Delta R\cdot \sin\left(\theta^{\prime }-\theta_{E}\right)\\ \cos\left(2\cdot \theta^{\prime }\right) \end{array}\right]}^{T}.$$

Substitution of (A5) into the first-order approximation (A3) yields the following expression for the magnetoresistive element conversion error:

(A6) $$\begin{array}{cc} e_{E}\left(\theta^{\prime }\right) & \approx r_{E,1}\\ & +r_{E,2}\cdot \cos\left(\theta^{\prime }-\theta_{E}\right)+r_{E,3}\cdot \Delta R\cdot \sin\left(\theta^{\prime }-\theta_{E}\right)\\ & +r_{E,4}\cdot \cos\left(2\cdot \theta^{\prime }\right). \end{array}$$

### Appendix A.2. Magnetoresistive Bridge Model Derivation

The total differential in the linearized bridge conversion-error model depends on the bridge configuration and excitation source. For a magnetoresistive measurement bridge powered by a constant-voltage source, partial differentiation of the bridge transfer function gives:

(A7) $$D_{R}u_{\mathrm{VB}}\left(\theta^{\prime }\right)=A\cdot V_{0}\cdot \left[\begin{array}{cccc} \frac{R_{3}}{{\left(R_{1}+R_{3}\right)}^{2}} & -\frac{R_{4}}{{\left(R_{2}+R_{4}\right)}^{2}} & -\frac{R_{1}}{{\left(R_{1}+R_{3}\right)}^{2}} & \frac{R_{2}}{{\left(R_{2}+R_{4}\right)}^{2}} \end{array}\right].$$

Evaluating this expression at the nominal operating point for the bridge with four active elements yields:

(A8) $$D_{R}u_{\mathrm{VB}}\left(\theta^{\prime }\right)=\frac{A\cdot V_{0}}{R}\cdot \left[\begin{array}{cccc} 1 & -1 & -1 & 1 \end{array}\right]-\frac{A\cdot V_{0}}{R^{2}}\cdot \Delta R_{E}\left(\theta^{\prime }\right).$$

Using the corresponding nominal bridge gain, the differential can be written in the simplified form:

(A9) $$D_{R}u_{\mathrm{VB}}\left(\theta^{\prime }\right)=K_{B}\cdot \left[\begin{array}{cccc} 1 & -1 & -1 & 1 \end{array}\right]-K_{B}\cdot \frac{\Delta R_{E}\left(\theta^{\prime }\right)}{R}.$$

For a magnetoresistive measurement bridge powered by a constant-current source, partial differentiation of the corresponding transfer function gives:

(A10) $$D_{R}u_{\mathrm{CB}}\left(\theta^{\prime }\right)=\frac{A\cdot I_{0}}{{\left(R_{1}+R_{2}+R_{3}+R_{4}\right)}^{2}}\cdot {\left[\begin{array}{c} \left(R_{2}+R_{4}\right)\cdot \left(R_{3}+R_{4}\right)\\ -\left(R_{1}+R_{3}\right)\cdot \left(R_{3}+R_{4}\right)\\ -\left(R_{1}+R_{2}\right)\cdot \left(R_{2}+R_{4}\right)\\ \left(R_{1}+R_{2}\right)\cdot \left(R_{1}+R_{3}\right) \end{array}\right]}^{T}.$$

Although the evaluation differs slightly for bridges with two and four active elements, substitution of the nominal values gives the same differential form at the nominal operating point:

(A11) $$D_{R}u_{\mathrm{CB}}\left(\theta^{\prime }\right)=\frac{K_{B}}{4}\cdot \left[\begin{array}{cccc} 1 & -1 & -1 & 1 \end{array}\right].$$

Comparison of (A9) and (A11) leads to the following common form of the total bridge-output differential at the nominal operating point:

(A12) $$\begin{array}{c} D_{R}u_{B}\left(\theta^{\prime }\right)=k_{1}+k_{2}\cdot \frac{\Delta R_{E}\left(\theta^{\prime }\right)}{R},\\ k_{1}=k_{1}\cdot K_{B}\cdot \left[\begin{array}{cccc} 1 & -1 & -1 & 1 \end{array}\right],\\ k_{2}=-k_{2}\cdot K_{B}\cdot \left[\begin{array}{cccc} 1 & 1 & 1 & 1 \end{array}\right]. \end{array}$$

To derive the harmonic representation of the bridge conversion error, let **r***_R_* denote the matrix whose columns contain the parameter-error vectors of the individual magnetoresistive elements:

(A13) $$r_{B}=\left[\begin{array}{cccc} r_{1}\;| & r_{2}\;| & r_{3}\;| & r_{4} \end{array}\right].$$

Using the first-order magnetoresistive-element error model, the vector of conversion errors of the bridge elements can be expressed in compact form as:

(A14) $$e_{R}\left(\theta^{\prime }\right)\approx {\left(D_{r}R_{E}\left(\theta^{\prime }\right)\cdot r_{B}\right)}^{T}=r_{B}^{T}\cdot {\left(D_{r}R_{E}\left(\theta^{\prime }\right)\right)}^{T}.$$

Substitution of this expression into the bridge conversion-error model gives:

(A15) $$e_{B}\left(\theta^{\prime }\right)\approx k_{2}\cdot r_{B}^{T}\cdot \underset{A^{*}}{\underset{︸}{{\left(D_{r}R_{E}\left(\theta^{\prime }\right)\right)}^{T}\cdot \frac{\Delta R_{E}\left(\theta^{\prime }\right)}{R}}}+k_{1}\cdot r_{B}^{T}\cdot \underset{B^{*}}{\underset{︸}{{\left(D_{r}R_{E}\left(\theta^{\prime }\right)\right)}^{T}}}.$$

Notice that only the auxiliary vectors **A*** and **B*** from (A15) are functions of the angle of spontaneous magnetization of the free ferromagnetic layer, while the remaining terms are constant parameters. Vector **B*** follows directly from the magnetoresistive-element linearization derived in Appendix A.1, while vector **A*** is obtained by applying the corresponding trigonometric transformations:

(A16) $$\begin{array}{cc} A^{*} & ={\left[\begin{array}{c} 1\\ \cos\left(\theta^{\prime }-\theta_{E}\right)\\ \Delta R\cdot \sin\left(\theta^{\prime }-\theta_{E}\right)\\ \cos\left(2\cdot \theta^{\prime }\right) \end{array}\right]}^{T}\cdot \frac{\Delta R}{R}\cdot \cos\left(\theta^{\prime }-\theta_{E}\right)\\ & =\frac{\Delta R}{R}\cdot \left[\begin{array}{c} \cos\left(\theta^{\prime }-\theta_{E}\right)\\ \cos^{2}\left(\theta^{\prime }-\theta_{E}\right)\\ \Delta R\cdot \sin\left(\theta^{\prime }-\theta_{E}\right)\cdot \cos\left(\theta^{\prime }-\theta_{E}\right)\\ \cos\left(2\cdot \theta^{\prime }\right)\cdot \cos\left(\theta^{\prime }-\theta_{E}\right) \end{array}\right]\\ & =\frac{\Delta R}{2R}\cdot \left[\begin{array}{c} 2\cdot \cos\left(\theta^{\prime }-\theta_{E}\right)\\ 1+\cos\left(2\cdot \theta^{\prime }-2\cdot \theta_{E}\right)\\ \Delta R\cdot \sin\left(2\cdot \theta^{\prime }-2\cdot \theta_{E}\right)\\ \cos\left(3\cdot \theta^{\prime }-\theta_{E}\right)+\cos\left(\theta^{\prime }+\theta_{E}\right) \end{array}\right]. \end{array}$$

The resulting harmonic functions and their corresponding coefficients are summarized in Table A1.

**Table A1:** Harmonic components of the auxiliary vectors A* and B*.

*i*	Order	*f_i_*(*θ*′)	*e_B,i_*
1	0	1	$k_{1}\cdot {\left[r_{i,1}\right]}^{T}+\frac{\Delta R}{R}\cdot \frac{k_{2}\cdot {\left[r_{i,2}\right]}^{T}}{2}$
2	1	cos(*θ*′ − *θ_E_*)	$k_{1}\cdot {\left[r_{i,2}\right]}^{T}+\frac{\Delta R}{R}\cdot k_{2}\cdot {\left[r_{i,1}\right]}^{T}$
3	1	sin(*θ*′ − *θ_E_*)	$\Delta R\cdot k_{1}\cdot {\left[r_{i,3}\right]}^{T}$
4	1	cos(*θ*′ + *θ_E_*)	$\frac{\Delta R}{R}\cdot \frac{k_{2}\cdot {\left[r_{i,4}\right]}^{T}}{2}$
5	2	cos(2 ⋅ *θ*′)	$k_{1}\cdot {\left[r_{i,4}\right]}^{T}$
6	2	cos(2 ⋅ *θ*′ − 2 ⋅ *θ_E_*)	$\frac{\Delta R}{R}\cdot \frac{k_{2}\cdot {\left[r_{i,2}\right]}^{T}}{2}$
7	2	sin(2 ⋅ *θ*′ − 2 ⋅ *θ_E_*)	$\frac{\Delta R^{2}}{R}\cdot \frac{k_{2}\cdot {\left[r_{i,3}\right]}^{T}}{2}$
8	3	cos(3 ⋅ *θ*′ − *θ_E_*)	$\frac{\Delta R}{R}\cdot \frac{k_{2}\cdot {\left[r_{i,4}\right]}^{T}}{2}$

Substitution of the harmonic functions from Table A1 into (A15) leads to the following harmonic representation of the bridge conversion error:

(A17) $$\begin{array}{cc} e_{B}\left(\theta^{\prime }\right) & \approx \sum_{i=1}^{8}e_{B,i}\cdot f_{i}\left(\theta^{\prime }\right)\\ & =\underset{\mathrm{order}=0}{\underset{︸}{e_{B,1}}}+\underset{\mathrm{order}=1}{\underset{︸}{\sum_{i=2}^{4}e_{B,i}\cdot f_{i}\left(\theta^{\prime }\right)}}+\underset{\mathrm{oreder}=2}{\underset{︸}{\sum_{i=5}^{7}e_{B,i}\cdot f_{i}\left(\theta^{\prime }\right)}}+\underset{\mathrm{oreder}=3}{\underset{︸}{e_{B,8}\cdot f_{8}\left(\theta^{\prime }\right)}}. \end{array}$$

For completeness, substituting the harmonic bridge conversion-error representation into the real measurement-bridge model yields:

(A18) $${\hat{u}}_{B}\left(\theta^{\prime }\right)=\left(1+g_{B}\right)\cdot K_{B}\cdot \Delta R_{S}\left(\theta^{\prime }\right)+\left(1+g_{B}\right)\cdot \left(\sum_{i=1}^{8}e_{B,i}\cdot f_{i}\left(\theta^{\prime }\right)+e_{0}\right).$$

The universal magnetoresistive-element characteristic can be expressed using the same harmonic basis as:

Δ*R_E_*(*θ*′) = Δ*R* ⋅ cos(*θ*′ + *θ_E_*) = Δ*R* ⋅ *f*_2_(*θ*′). (A19)

Finally, substituting (A19) into (A18) yields the following model of the magnetoresistive measurement bridge:

(A20) $$\begin{array}{cc} {\hat{u}}_{B}\left(\theta^{\prime }\right) & =\left(1+g_{B}\right)\cdot K_{B}\cdot \Delta R\cdot f_{2}\left(\theta^{\prime }\right)+\left(1+g_{B}\right)\cdot \left(\sum_{i=1}^{8}e_{B,i}\cdot f_{i}\left(\theta^{\prime }\right)+e_{0}\right)\\ & =\underset{\mathrm{order}=0}{\underset{︸}{\left(1+g_{B}\right)\cdot \left(e_{B,1}+e_{0}\right)}}+\underset{\mathrm{order}=1}{\underset{︸}{\left(1+g_{B}\right)\cdot \left(K_{B}\cdot \Delta R\cdot f_{2}\left(\theta^{\prime }\right)+\sum_{i=2}^{4}e_{B,i}\cdot f_{i}\left(\theta^{\prime }\right)\right)}}\\ & +\underset{1<\mathrm{order}\leq 3}{\underset{︸}{\left(1+g_{B}\right)\cdot \sum_{i=5}^{8}e_{B,i}\cdot f_{i}\left(\theta^{\prime }\right)}}. \end{array}$$

The resulting magnetoresistive measurement-bridge model is a harmonic function of the free ferromagnetic layer magnetization angle containing harmonics up to the third order.

### Appendix A.3. Derivation of the Residual Magnetic-Field Angle Error

The universal model of the magnetic field direction sensor can be expressed as:

(A21) $$\begin{array}{c} \phi^{\prime }=m_{B}\cdot \phi ,\\ \hat{u}\left(\phi^{\prime }\right)=G\cdot \left[\begin{array}{c} \cos\left(\phi^{\prime }\right)\\ \sin\left(\phi^{\prime }\right) \end{array}\right]+o+e\left(\phi^{\prime }\right). \end{array}$$

To express the measurement result directly in terms of the magnetic field angle, the residual error of the measuring bridges ***e***(*ϕ*) is replaced by the residual magnetic-field angle error $\hat{e}$ (*ϕ*′), resulting in the equivalent representation:

(A22) $$\begin{array}{c} \phi^{\prime }=m_{B}\cdot \phi ,\\ \hat{u}\left(\phi^{\prime }\right)=G\cdot \left[\begin{array}{c} \cos\left(\phi^{\prime }+m_{B}\cdot \hat{e}\left(\phi^{\prime }\right)\right)\\ \sin\left(\phi^{\prime }+m_{B}\cdot \hat{e}\left(\phi^{\prime }\right)\right) \end{array}\right]+o. \end{array}$$

Since (A21) and (A22) describe the same sensor output, equating the two expressions gives:

(A23) $$G\cdot \left[\begin{array}{c} \cos\left(\phi^{\prime }\right)\\ \sin\left(\phi^{\prime }\right) \end{array}\right]+o+e\left(\phi^{\prime }\right)=G\cdot \left[\begin{array}{c} \cos\left(\phi^{\prime }+m_{B}\cdot \hat{e}\left(\phi^{\prime }\right)\right)\\ \sin\left(\phi^{\prime }+m_{B}\cdot \hat{e}\left(\phi^{\prime }\right)\right) \end{array}\right]+o.$$

Rearranging (A23) yields:

(A24) $$\left[\begin{array}{c} \cos\left(\phi^{\prime }\right)\\ \sin\left(\phi^{\prime }\right) \end{array}\right]+G^{-1}\cdot e\left(\phi^{\prime }\right)=\left[\begin{array}{c} \cos\left(\phi^{\prime }+m_{B}\cdot \hat{e}\left(\phi^{\prime }\right)\right)\\ \sin\left(\phi^{\prime }+m_{B}\cdot \hat{e}\left(\phi^{\prime }\right)\right) \end{array}\right].$$

Taking the argument of both sides of (A24) gives the following implicit expression for the residual magnetic-field angle error $\hat{e}$ (*ϕ*′):

(A25) $$\arg\left\{\left[\begin{array}{c} \cos\left(\phi^{\prime }\right)\\ \sin\left(\phi^{\prime }\right) \end{array}\right]+G^{-1}\cdot e\left(\phi^{\prime }\right)\right\}=\phi^{\prime }+m_{B}\cdot \hat{e}\left(\phi^{\prime }\right).$$

To obtain an explicit approximate expression for $\hat{e}$ (*ϕ*′), (A25) is linearized around the nominal operating point. The gradient of the argument vector is given by:

(A26) $$\nabla \arg\left\{\left[\begin{array}{c} x\\ y \end{array}\right]\right\}=\frac{1}{x^{2}+y^{2}}\left[\begin{array}{c} -y\\ x \end{array}\right],$$

Applying the first-order approximation around the nominal operating point gives:

(A27) $$\begin{array}{ll} \arg\left\{\left[\begin{array}{c} \cos\left(\phi^{\prime }\right)\\ \sin\left(\phi^{\prime }\right) \end{array}\right]+G^{-1}\cdot e\left(\phi^{\prime }\right)\right\} & \approx \arg\left\{\left[\begin{array}{c} \cos\left(\phi^{\prime }\right)\\ \sin\left(\phi^{\prime }\right) \end{array}\right]\right\}+\nabla \arg{\left\{\left[\begin{array}{c} \cos\left(\phi^{\prime }\right)\\ \sin\left(\phi^{\prime }\right) \end{array}\right]\right\}}^{T}\cdot G^{-1}\cdot e\left(\phi^{\prime }\right)\\ & =\phi^{\prime }+{\left[\begin{array}{c} -\sin\left(\phi^{\prime }\right)\\ \cos\left(\phi^{\prime }\right) \end{array}\right]}^{T}\cdot G^{-1}\cdot e\left(\phi^{\prime }\right). \end{array}$$

Finally, substituting (A27) into (A25) and rearranging, the residual magnetic-field angle error is approximately given by:

(A28) $$\hat{e}\left(\phi^{\prime }\right)\approx m_{B}^{-1}\cdot {\left[\begin{array}{c} -\sin\left(\phi^{\prime }\right)\\ \cos\left(\phi^{\prime }\right) \end{array}\right]}^{T}\cdot G^{-1}\cdot e\left(\phi^{\prime }\right).$$

Since the gain matrix ***G*** is constant and the measuring-bridge residual error contains harmonics of at most the third order, (A28) shows that the residual magnetic-field angle error is limited to harmonics of at most the fourth order.
